# A novel multi-modal rehabilitation monitoring over human motion intention recognition

**DOI:** 10.3389/fbioe.2025.1568690

**Published:** 2025-07-17

**Authors:** Saleha Kamal, Mohammed Alshehri, Yahya AlQahtani, Abdulmonem Alshahrani, Nouf Abdullah Almujally, Ahmad Jalal, Hui Liu

**Affiliations:** ^1^ Jiangsu Key Laboratory of Intelligent Medical Image Computing, School of Artificial Intelligence, Nanjing University of Information Science and Technology, Nanjing, China; ^2^ Faculty of Computer Science and AI, Air University, E‐9, Islamabad, Pakistan; ^3^ Department of Computer Science, Applied College, King Khalid University, Abha, Saudi Arabia; ^4^ Department of Informatics and Computer Systems, King Khalid University, Abha, Saudi Arabia; ^5^ Department of Information Systems, College of Computer and Information Sciences, Princess Nourah bint Abdulrahman University, Riyadh, Saudi Arabia; ^6^ Department of Computer Science and Engineering, College of Informatics, Korea University, Seoul, Republic of Korea; ^7^ Guodian Nanjing Automation Co., Ltd., Nanjing, China; ^8^ Cognitive Systems Lab, University of Bremen, Bremen, Germany

**Keywords:** motion intension recognition, human machine interaction, rehabilitation, multimodal sensor integration motion intension recognition, multimodal sensor integration

## Abstract

Human Motion Intention Recognition (HMIR) plays a vital role in advancing medical rehabilitation and assistive technologies by enabling the early detection of pain-indicative actions such as sneezing, coughing, or back discomfort. However, existing systems struggle with recognizing such subtle movements due to complex postural variations and environmental noise. This paper presents a novel multi-modal framework that integrates RGB and depth data to extract high-resolution spatial-temporal and anatomical features for accurate HMIR. Our method combines kinetic energy, optical flow, angular geometry, and depth-based features (e.g., 2.5D point clouds and random occupancy patterns) to represent full-body dynamics robustly. Stochastic Gradient Descent (SGD) is employed to optimize the feature space, and a deep neuro-fuzzy classifier is proposed to balance interpretability and predictive accuracy. Evaluated on three benchmark datasets—NTU RGB + D 120, PKUMMD, and UWA3DII—our model achieves classification accuracies of 94.50%, 91.23%, and 88.60% respectively, significantly outperforming state-of-the-art methods. This research lays the groundwork for future real-time HMIR systems in smart rehabilitation and medical monitoring applications.

## 1 Introduction

Human Motion Intention Recognition (HMIR) stands as a revolutionary computing domain that utilizes sophisticated computational models to interpret human movements (Ahmad et al., 2020). The applications of HMIR systems continue to broaden throughout surveillance security and human-computer interaction, but healthcare remains their most critical domain of deployment (Mahwish et al., 2021). HMIR shows remarkable potential to detect subtle medical movements such as sneezing and coughing while recognizing signals from back distress and neck discomfort and other specific regional pain indicators. Successful medical HMIR systems require a multi-modal architecture which combines RGB along with depth data for enhanced detection of hard-to-detect movements (Azmat and Ahmad, 2021).

HMIR stands as a revolutionary computing domain that utilizes sophisticated computational models to interpret human movements. The applications of HMIR systems continue to broaden throughout surveillance security and human-computer interaction but healthcare remains their most critical domain of deployment (M. Muneeb et al., 2023). HMIR shows remarkable potential to detect subtle medical movements such as sneezing and coughing while recognizing signals from back distress and neck discomfort and other specific regional pain indicators. Successful medical HMIR systems require a multi-modal architecture which combines RGB along with depth data for enhanced detection of hard-to-detect movements (Mahwish and Ahmad, 2023).

Applications of a multi-modal HMIR framework include RGB and depth data to achieve complete spatial and temporal details. When RGB data combines with depth data it delivers detailed visual information that is enhanced by depth details which reduce the impact of scene lighting variations and spatial occlusions (Amir et al., 2020a). The integrated system produces enhanced human action recognition capabilities which work especially well in medical settings with complex environments and poor lighting conditions (Ahmad et al., 2019).

The proposed approach leverages three key datasets—NTU RGB + D 120, PKUMMD, and UWA3DII—known for their diversity in activities and environments. These datasets which feature comprehensive patient scenarios validate model training because they expand medical application possibilities. Through the combination of RGB and depth data extraction techniques the HMIR system obtains both detailed spatial information and temporal pattern analysis. Kinetic energy alongside Histograms of Optical Flow (HOF) and angular geometric features together with eight round angles create the RGB features for analyzing movement dynamics and postural modifications. Depth features incorporate 2.5D point clouds together with random occupancy patterns and movement polygon which add to RGB data by providing strong three-dimensional spatial understanding to resist occlusions and environmental changes. The performance optimization process depends on stochastic gradient descent (SGD) for achieving efficient and accurate model convergence (Ogbuabor et al., 2018). A neuro-fuzzy classifier performs classification work by aligning neural network adaptability with fuzzy logic interpretability for identifying medical significant movements with precision. Our proposed research contributions to this field:

• This study introduces a multi-modal approach combining RGB and depth data to extract features like kinetic energy, HOF, angular geometry, and 3D spatial patterns, enabling precise analysis of human activities in medical contexts
• The proposed system incorporates stochastic gradient descent (SGD) for optimization, ensuring rapid convergence and robustness even when handling large-scale datasets, which are essential for real-world applications.
• By employing a neuro-fuzzy classifier, the research addresses the challenges of uncertainty and overlapping characteristics in medical activity recognition. This classifier enhances interpretability and precision, ensuring accurate differentiation of subtle actions.
• A comprehensive benchmark evaluation of the model takes place on NTU RGB + D 120, H3D and PKUMMD datasets to assess its performance for different medical applications. The proposed model exceeds all current benchmark methods by delivering superior outcomes for accuracy, precision, F1 score and recall metrics.

The remainder of this paper is structured as follows: Section 2 provides a comprehensive review of existing literature in the domain of Human Motion Intention Recognition (HMIR), particularly focusing on multimodal approaches and rehabilitation applications. Section 3 details the materials and methodology, including system architecture, preprocessing, segmentation, skeleton generation, and feature extraction techniques. Section 4 presents the feature optimization and classification techniques used, including stochastic gradient descent and the deep neuro-fuzzy classifier. Section 5 describes the performance evaluation setup and elaborates on the benchmark datasets used. Section 6 discusses experimental results, learning curves, and ablation studies, followed by a comparative analysis with state-of-the-art methods. Section 7 outlines the implications, potential applications, and limitations of the proposed system. Finally, Section 8 concludes the paper and suggests directions for future research.

## 2 Literature review

The study by Chen et al. (2021) developed a hybrid vision-based system that employed RGB and depth sensors to track stroke patient rehabilitation activities. The system processed depth sensor skeleton features together with RGB video spatiotemporal data to produce its inputs. A dual-stream convolutional neural network analyzed multiple input modalities to achieve 91% success in detecting rehabilitation exercises. The system showed drawbacks because outdoor lighting fluctuations deteriorated the quality of RGB sensor information. The study conducted by Lin et al. (2023) investigated depth data interpretation for Human Motion Intention Recognition (HMIR) in assistive technologies. Their research developed a HMIR model with depth sensors that traced joint movements to generate advanced motion pathway data for people who need help walking. The developed model performed better than traditional depth-only approaches achieving a fall detection accuracy with an F1 score of 0.94. The system faced persistent problems detecting occlusions when operating in crowded areas (Riedel et al., 2008).

A multimodal fusion approach was developed by Xefteris et al. (2024) to merge inertial sensors with an RGB camera for 3D human pose analysis in posture correction therapy. The hybrid LSTM-Random Forest fusion network operated on time-series motion data to generate accurate improper movement detection outcomes. The system needed extensive computational capacity which made its real-time deployment impractical. The research team of Jang et al. (2020) developed an assistive system powered by RGB-D sensors to track daily routines of elderly individuals at affordable prices. The ETRI-Activity3D dataset provided researchers with real-world daily elderly life action categories alongside tools to analyze large-scale activity recognition problems. The system monitored body positions with depth information as it aimed to create budget-friendly monitoring solutions that avoided invasive medical procedures. Sensor blocks and individual behavioral variations led to detection challenges for the system when monitoring subtle movements.

A system for human activity detection utilizing encrypted RGB and depth data was developed by Wang et al. (2022). The study demonstrated that real-time functionality could run smoothly when privacy protection protocols were implemented. Data encryption created restrictions that diminished the system’s measurement precision. A detection system for fainting events in elderly populations was created by depth sensors using their ability to analyze skeletal changes (Chalvatzaki et al., 2018). The combination of long short-term memory cells within the network produced accurate human gait stability predictions by analyzing fainting events. Despite these shortcomings the system showed reduced effectiveness for detecting subtle or incomplete transitions that could signal potential syncope events requiring further development.

An RGB-D-based system created by Chen and Fisher (2023) monitored older adult inactivity through detecting prolonged periods of immobility that indicated potential health issues. The lightweight camera monitoring system delivered successful results across multiple environments although issues with irregular lighting conditions and obstructed objects negatively affected its operational stability. (Elforaici et al. (2018) invented a rehabilitation monitoring system that used RGB cameras to measure repetitive motion as well as posture asymmetry. This technique demonstrated average accuracy because it processed only RGB information yet shown flexibility to illumination changes although it failed to capture depth perception.

The researchers developed a real-time fall detection system using depth sensors which found applications in medical settings according to Smith et al. (2021). The model showed high sensitivity that led to quick emergency responses. System performance suffered due to its inability to distinguish abrupt non-critical movements during activities such as sitting from genuine accidental falls. An integrated fainting detection solution was proposed by Huang et al. (2020) through combining depth sensors with thermal sensors. Strong environmental adaptability combined with excellent operational performance enabled the system to detect fainting incidents effectively. Additional system modifications were required to make the system more sensitive yet still maintain total reliability standards (Rodrigues et al., 2022).

## 3 Materials and methods

### 3.1 System methodology

The developed framework effectively obtains distinct temporal and spatial features from RGB images and depth data to establish robust activity group classification. The system transforms video frames into images to execute preprocessing activities that remove unnecessary data while obtaining crucial information. The system maintains high data quality while achieving efficient computation through its data processing sequence. The preprocessing phase consists of three key steps: image normalization, noise removal, and Region of Interest (ROI) extraction. Through a combination of image normalization and noise removal processes the data achieves enhanced refinement before ROI extraction produces distinct vital regions by filtering away nonessential background components. Body segmentation techniques enable more effective subject isolation by disassociating human figures from their surrounding environments. The determination of subjects becomes precise when depth data is used accurately throughout this processing stage because it yields detailed boundary definitions for improved subsequent analytical results. The system conducts skeleton extraction and key-point generation procedures which derive vital spatial information from segmented contours.

**FIGURE 1:**
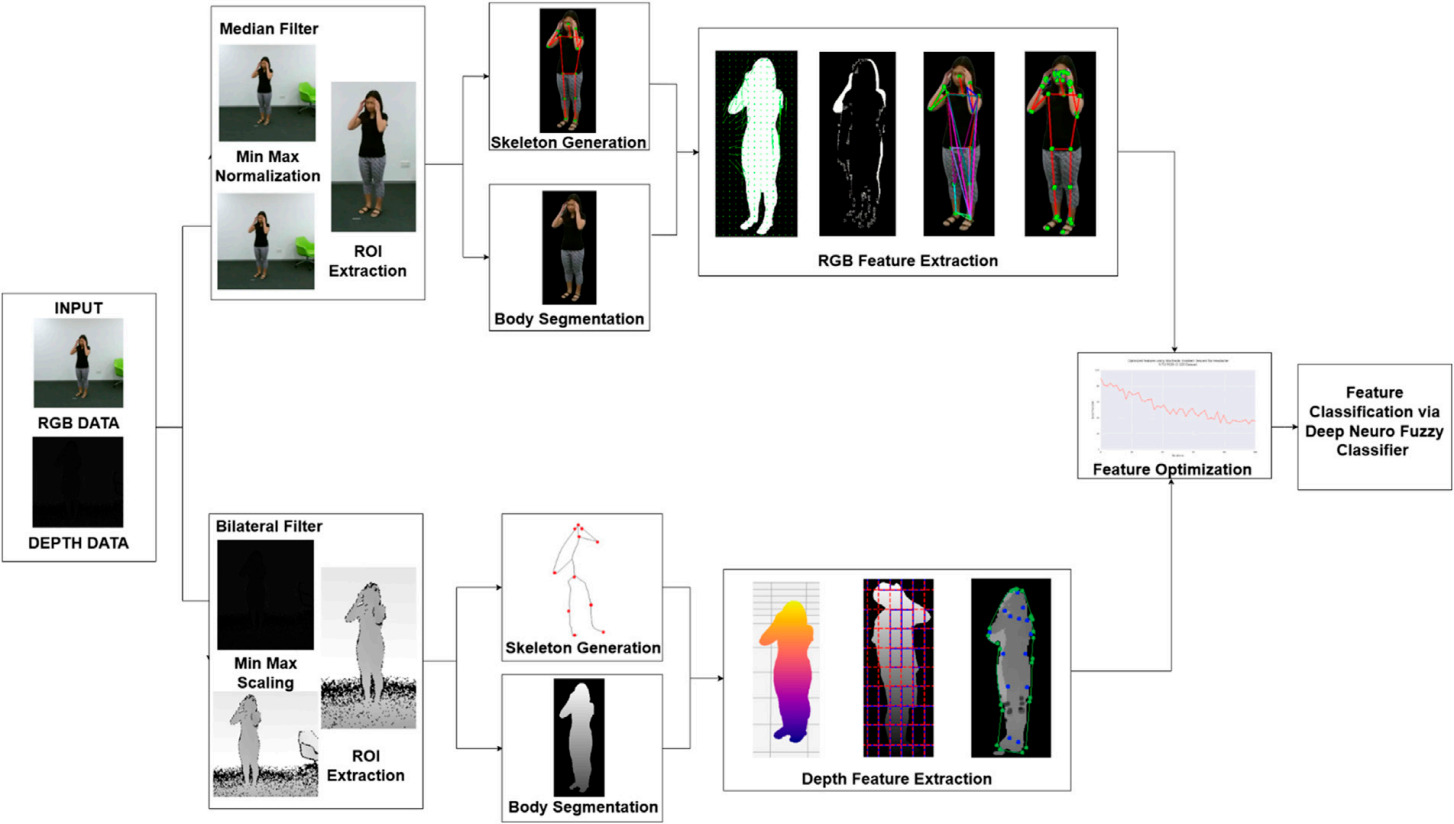
The architecture of the proposed system.

The system employs a method which reduces extracted features to optimize performance versus precision in identification tasks. The optimized features travel to a classifier for both accurate and efficient activity recognition. Figure 1 shows a complete model design that combines RGB and depth data to boost HMIR performance. This multi-modal approach leverages the complementary strengths of both data types, resulting in a robust system capable of accurately identifying subtle and complex human activities.

### 3.2 Image preprocessing

Image preprocessing plays a pivotal role in handling video or image-based data, as it helps eliminate irrelevant information while enhancing image quality for seamless and efficient analysis. This stage involves three primary steps: 1) Normalization, 2) Noise reduction, and 3) ROI identification. For RGB data, video sequences are first converted into individual image frames, with the number of frames per video varying significantly. Given that typical videos contain around 30 frames per second, processing all frames can lead to increased system complexity (Nida et al., 2021). To address this, we reduced the frame count to 20 keyframes by analyzing pixel intensity through histogram distribution and selecting frames with the most significant intensity variations. Mathematically expressed in Equation 1:

$$H\left(I\right)={\arg\max}_{i\in \left(1,\ldots ,N\right)}\left(\sum_{k=1}^{B}\sqrt{{\left(H_{i}\left(k\right).H_{i-1}\left(k\right)\right)}^{2}}\right)$$ (1)

where $H_{i}\left(k\right)$ represents the *kth* bin in the histogram for the *ith* time frame. *N* is the total number of frames and *B* is the total number of bins in the histogram. Once the frames are extracted from the RGB and depth data, the next step involves removing noise to enhance image quality while preserving essential details for subsequent analysis. To address the unique characteristics of each modality, we applied separate denoising techniques: median filtering for RGB data and bilateral filtering for depth data. Median filtering is a non-linear approach particularly effective for removing salt-and-pepper noise in RGB images. In this method, each pixel is replaced with the median of its neighboring pixel intensities within a predefined window. By focusing on the central tendency of pixel values, median filtering not only eliminates noise but also preserves edges, which are critical for accurate feature extraction. Mathematically expressed in Equation 2:

$$M\left[v\right]\left(i,j\right)=median\left\{v\left(x,y\right)|\left(x,y\right)\in N\left(i,j\right)\right\}$$ (2)

where $M\left[v\right]\left(i,j\right)$ is the median filtered value at pixel $\left(i,j\right)$ , $v\left(x,y\right)$ is the original pixel value at position $\left(x,y\right)$ and $N\left(i,j\right)$ is the neighborhood of pixel $\left(i,j\right)$ defined by the kernel size. Bilateral filtering performs depth data processing in a manner that maintains all depth discontinuities without compromising their effectiveness (Raza et al., 2023). The bilateral filtering mechanism applies weighted pixels averaging through which distance between pixels and their intensity match levels impact weight intensity. Spatial and intensity data combine to make pixels that are closer to both factors receive greater importance in the averaging mechanism (Min et al., 2020). Mathematically, the bilateral filter is defined in Equation 3:

$$B\left[v\right]\left(i,j\right)=\frac{1}{W\left(i,j\right)}\sum_{\left(x,y\right)\in N\left(i,j\right)}v\left(x,y\right)\cdot \exp\left(-\frac{{\left\|\left(i,j\right)-\left(x,y\right)\right\|}^{2}}{2\sigma_{s}^{2}}-\frac{{\left\|v\left(i,j\right)-v\left(x,y\right)\right\|}^{2}}{2\sigma_{r}^{2}}\right)$$ (3)

where $B\left[v\right]\left(i,j\right)$ represents the filtered value at pixel $\left(i,j\right)$ , $v\left(x,y\right)$ is the intensity of the pixel at location $\left(x,y\right)$ , and $N\left(i,j\right)$ denotes the neighborhood around pixel $\left(i,j\right)$ . The parameters $\sigma_{s}^{2}$ apply influence on both pixel distance characteristics from others and pixel intensity similarity features. The normalization factor $W\left(i,j\right)$ ensures that the weights sum to one. This filtering technique uses these parameters to produce smoothed depth data which protects essential structural boundaries needed for depth assessments. The joint implementation of median filtering for RGB data with bilateral filtering for depth information achieves optimal denoising results for each modality (Mushhood et al., 2023).

Uniform pixel value scales across all images become the focus during image normalization subsequent to denoising. Image normalization stands as an essential step because both model performance and generalization quality benefit from it significantly. Without normalization the learning process becomes controlled by features with large values that lead to inaccurate predictions. Different normalization approaches exist in architecture which support the preservation of distinct features between RGB images and depth images (S. Hafeez et al., 2021). Dimension normalization techniques function differently between RGB images where Min-Max normalization is used and depth images which require Z-Score normalization.

Through Min-Max normalization the pixel values in RGB data acquire values between 0 and 1 while preserving their initial relative strength patterns across the data set. Mathematically, this technique is expressed as follows in Equation 4:

$$M\left(v\right)=\frac{I\left(x,y\right)-\mathrm{Min}}{\mathrm{Max}-\mathrm{Min}}$$ (4)

In the above equation, $I\left(x,y\right)$ represents the original pixel value at position $\left(x,y\right)$ , while Max and Min denote the maximum and minimum pixel values of the image, respectively. The normalization method through value scaling produces consistent data while lowering the impact of fluctuating light conditions in RGB image bases (Saleha and Jalal, 2024).

Z-Score normalization shifts depth pixel values to zero mean through scaling that depends on standard deviation. The method provides advanced depth information processing by managing the variations in scene intensity distribution patterns (Singh et al., 2022). Z-Score normalization is expressed in Equation 5:

$$Z\left(v\right)=\frac{I\left(x,y\right)-\mu }{\sigma }$$ (5)

In this equation, $I\left(x,y\right)$ is the pixel value at position $\left(x,y\right)$ , μ shows the mean pixel value of the image, and σ depicts the standard deviation of pixel values. Conducting this transformation produces data with 0 mean and 1 standard deviation thus making the data ready for depth measurements which require extensive value ranges between frames.

The ROI extraction function operates as the final step of preprocessing (Hammad et al., 2022). The selection of regions of interest enables researchers to pinpoint image sections containing human shapes which forms a critical step in the process. The system accuracy and computational simplicity increase when ROI focuses analysis on particular image sections. The proposed design utilizes an automated method to extract target regions from RGB and depth data which maintains accurate and consistent region detection throughout multiple modalities.

Connected component analysis serves as the method for extracting ROI by detecting human silhouettes. The method arranges neighboring pixels with equivalent brightness levels to recognize objects present in the image. After component detection the algorithm calculates dimensions to create bounding boxes around identified regions (Mushhood et al., 2022). Mathematically, connected components can be represented in Equation 6:

$$C_{i}=\left\{\left(x_{i},y_{i},w_{i},h_{i}\right)|\sum_{p=x_{i}}^{x_{i}+w_{i}}\sum_{q=y_{i}}^{y_{i}+h_{i}}f\left(p,q\right)>0\right\}$$ (6)

Here, $C_{i}$ represents a connected component, $x_{i}$ and $y_{i}$ denotes the coordinates of the top-left corner of the bounding box, and $w_{i}$ and $h_{i}$ are the width and height of the bounding box, respectively. The double summation $\sum_{p=x_{i}}^{x_{i}+w_{i}}\sum_{q=y_{i}}^{y_{i}+h_{i}}f\left(p,q\right)$ ensures that the bounding box encloses all pixels $\left(p,q\right)$ where the intensity function $f\left(p,q\right)$ is greater than zero, indicating the presence of relevant components. Once the connected components are computed, bounding boxes are drawn around the detected human silhouettes to define the ROI. The extracted ROI can be mathematically represented in Equation 7:

$${ROI}_{i}\left(x,y\right)=I\left(x,y\right)\;for\;x_{i}\leq x\;<\;x_{i}+w_{i},y_{i}\leq y\;<\;y_{i},h_{i}$$ (7)

**FIGURE 2:**
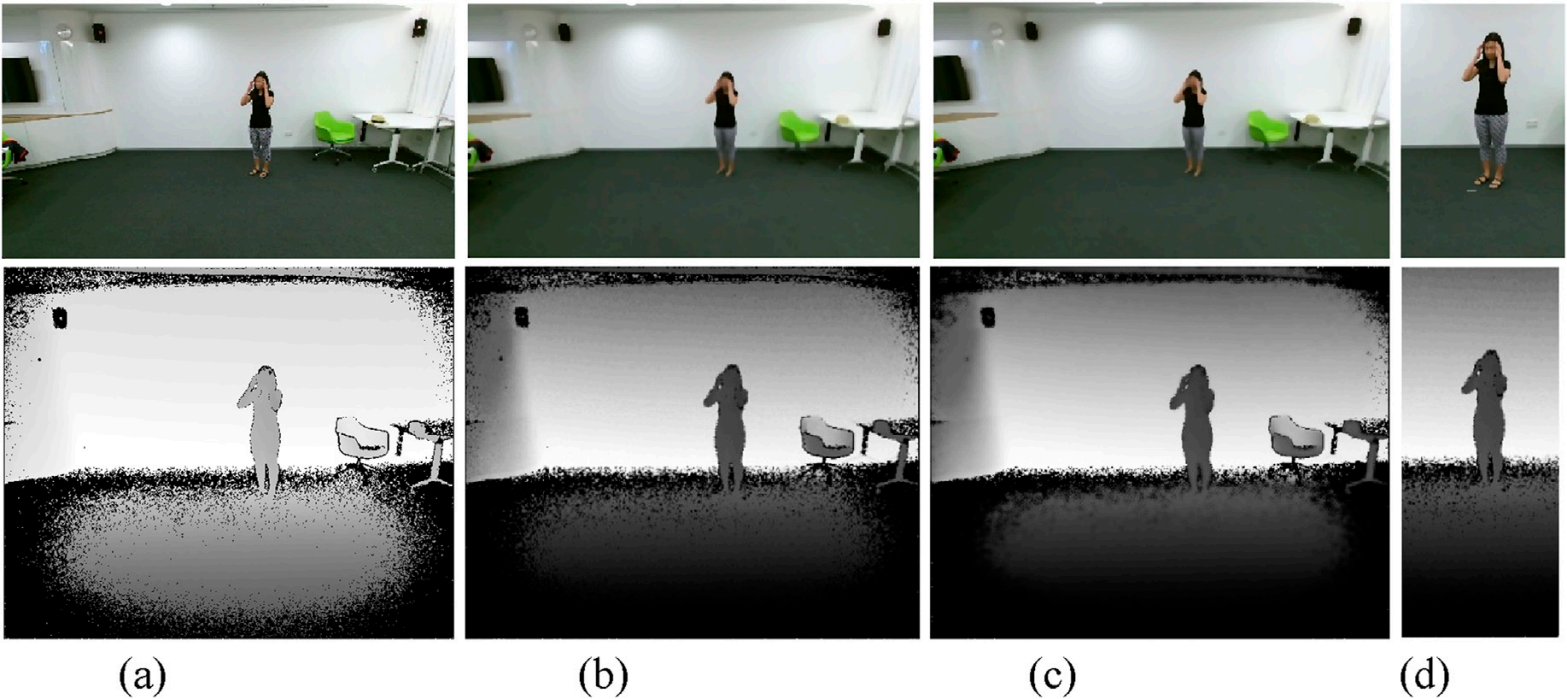
Visuals of Preprocessing pipeline **(a)** Original Frames **(b)** Noise Removal **(c)** Image Normalization **(d)** ROI Extraction on NTU RGB + D 120 dataset.

Here, ${ROI}_{i}\left(x,y\right)$ represents the pixel values within the extracted region of interest, and $I\left(x,y\right)$ corresponds to the pixel values of the original image. This formulation ensures that only the relevant portions of the image containing the human silhouette are retained for further processing. The automated ROI extraction method efficiently processes both RGB and depth data by dynamically identifying and isolating human silhouettes in varying conditions. Figure 2 illustrates the preprocessing pipeline for both RGB and depth data (Rafiq et al., 2024).

### 3.3 Body segmentation

In computer vision applications body segmentation proves essential for human interaction recognition because it creates effective boundaries between human figures and their environmental contexts (Poulose et al., 2021). Segmentation creates a human-focused section by removing extraneous background features to allow enhanced analytical accuracy and speed. Silhouettes act as fundamental image elements while all surrounding video content functions as background information. Segmentation serves an essential role by extracting meaningful features which lead to improved classification system accuracy. The work uses level set segmentation for RGB data processing while combining RGB and depth-based methods for depth data to produce exact and reliable silhouette extraction across both data types.

For RGB data, body segmentation was achieved using level set segmentation. In this method, the silhouette is represented implicitly using a level set function, $\phi \left(x,y,t\right)$ , where the zero level set ϕ=0 corresponds to the boundary of the object. The evolution of the contour is governed by the minimization of an energy function that integrates internal forces to ensure smoothness and external forces to attract the contour to object boundaries (Fatima et al., 2024). The energy functional is mathematically represented in Equation 8:

$$E_{total}=\int_{\Omega }\left(\mu {\left|\nabla \left.\phi \right|\right.}^{2}+\lambda g\left(I\right)\left|\nabla \phi \right|+v\;H\left(\phi \right)\right)dxdy$$ (8)

**FIGURE 3:**
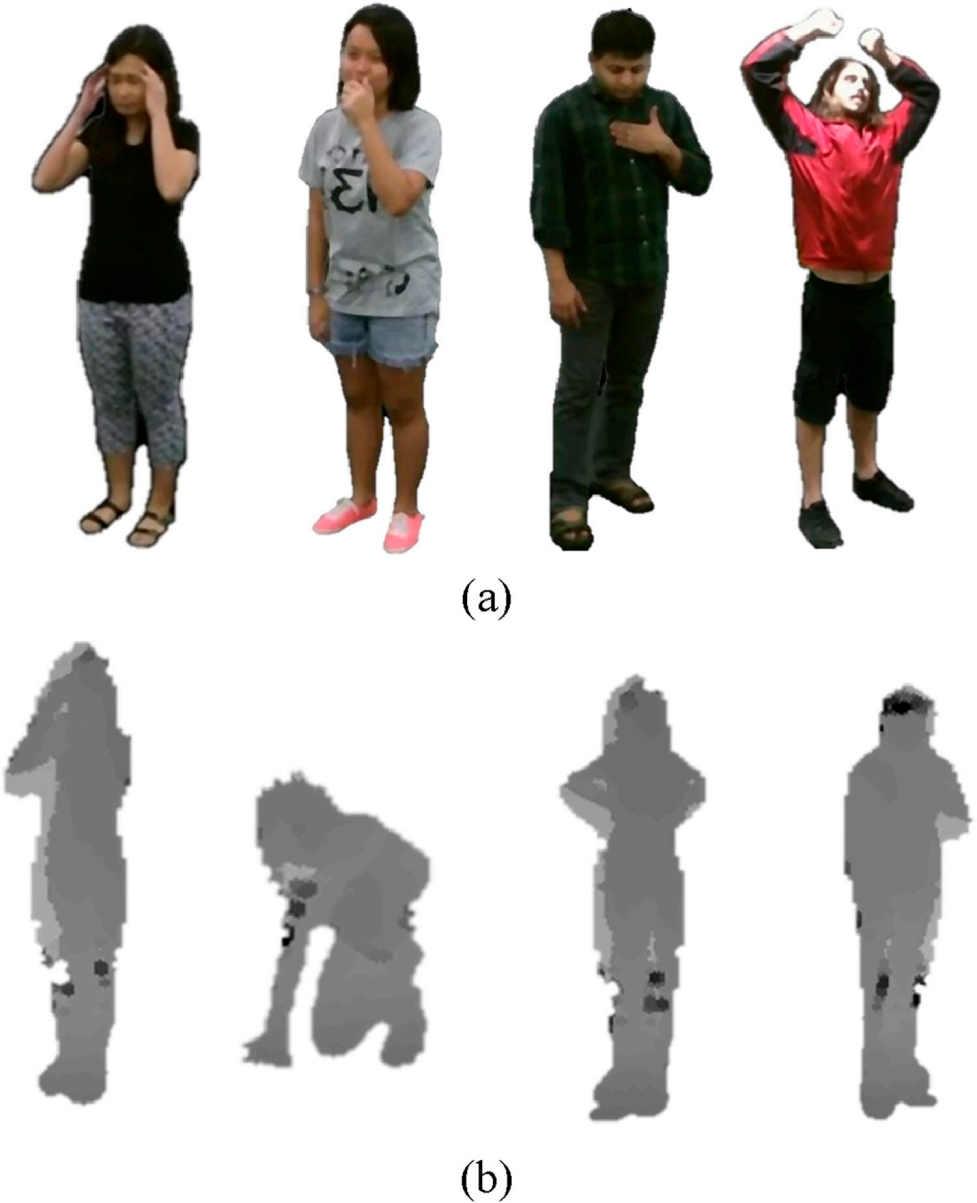
Illustration of Body Segmentation on RGB and Depth frames **(a)** Cough Falling Down Back Pain Fan self on NTU RGB + D 120 dataset. Illustration of Body Segmentation on Depth frames **(b)** Cough Falling Down Back Pain Fan self on NTU RGB+D 120 dataset.

Here, μ,λ and v are weighting parameters that control the influence of each energy component. The first term, ${\left|\nabla \left.\phi \right|\right.}^{2}$ , penalizes irregularities in the contour, ensuring smooth evolution. The second term involves $g\left(I\right)$ , an edge indicator function given in Equation 9:

$$g\left(I\right)=\frac{1}{1+{\left|\nabla \left.I\right|\right.}^{2}}$$ (9)

where $\left|\nabla \left.I\right|\right.$ represents the gradient magnitude of the image intensity, directing the contour toward high-gradient regions. The third term, $H\left(\phi \right)$ , is the Heaviside function, which controls the contour’s size and position. The evolution of the level set function is expressed by the following partial differential Equation 10:

$$\frac{\partial \phi }{\partial t}=\mu \cdot \kappa +\lambda g\left(I\right)\left.\cdot \right|\nabla \left.\phi \right|-v$$ (10)

where $\kappa =\mathrm{div}\left(\frac{\nabla \phi }{\left.\left|\nabla \right.\phi \right|}\right)$ is the curvature of the contour, ensuring smoothness. The term $g\left(I\right)$ attracts the contour to object edges, while −v serves as a balloon force, adjusting the contour’s size. In the proposed system, level set segmentation begins with an initial contour placed around the approximate boundary of the human silhouette. The contour iteratively adapts to align with the true boundaries of the silhouette based on the gradient and curvature information (Muhammad Hamdan and Ahmad, 2024). This approach ensures accurate isolation of the human silhouette, effectively separating the foreground from the background as illustrated in Figure 3.

Unlike RGB data, Depth data provides rich spatial information, making it effective for capturing the structure and posture of human figures. In this approach, affine transformations serve as the primary technique for segmenting depth silhouettes by aligning them spatially and geometrically within a consistent reference frame. This approach ensures precise segmentation and eliminates discrepancies caused by variations in perspective or acquisition conditions (Iqra and Ahmad, 2024).

Affine transformation is a linear mapping method that preserves geometric properties such as points, straight lines, and planes while enabling translation, scaling, rotation, and shearing. It is mathematically represented in Equation 11:

$$\left[\begin{array}{c} x^{\prime }\\ y^{\prime } \end{array}\right]=\left[\begin{array}{cc} a & b\\ c & d \end{array}\right]\left[\begin{array}{c} x\\ y \end{array}\right]+\left[\begin{array}{c} t_{x}\\ t_{y} \end{array}\right]$$ (11)

In this equation, $\left(x,y\right)$ are the original pixel coordinates in the depth image, and $\left(x^{\prime },y^{\prime }\right)$ represent the transformed coordinates. The parameters a,b,c,d constitute the transformation matrix, encapsulating scaling, rotation, and shearing operations. While $t_{x}$ and $t_{y}$ define translation components. The system computes a transformation matrix based on depth image silhouette spatial features to achieve alignment with a predefined reference region.

Segmentation starts when the affine transformation aligns depth data for the target reference frame. The alignment process follows detected high gradient magnitude regions on depth maps which represent human silhouette edges. The gradient magnitude is expressed in Equation 12:

$$|\nabla D\left(x,y\right)\left|=\right.\sqrt{{\left(\frac{\partial D}{\partial x}\right)}^{2}+{\left(\frac{\partial D}{\partial y}\right)}^{2}}$$ (12)

where $D\left(x,y\right)$ denotes the depth value at pixel $\left(x,y\right)$ . The computed gradient highlights areas of significant depth transitions, enabling accurate boundary localization. Once the affine transformation aligns the silhouette within the reference frame, a binary mask is applied to isolate the region of interest, effectively separating the foreground (human silhouette) from the background (Tayyab and Ahmad, 2024).

This technique ensures that the segmented depth silhouette remains spatially consistent and free from misalignments, thereby providing a clear and accurate representation of the human figure. To achieve this, affine transformation is employed as a robust and mathematically precise approach for segmenting depth data. Figure 3 illustrates depth datasegmentaion effectively.

### 3.4 Skeleton and key point generation

Skeleton extraction is a crucial step in pose estimation and human movement analysis, allowing for the identification and structured representation of key body landmarks. By extracting skeletal features, it becomes possible to analyze motion patterns, assess posture, and understand body mechanics—essential aspects for applications in sports science, healthcare, and animation. This approach simplifies the complexity of human motion by focusing on connections between major joints, providing a concise yet informative depiction of the human form (Fakhra and Ahmad, 2024).

In this work, MediaPipe Pose was selected due to its proven high accuracy in landmark detection, achieving a normalized landmark error of less than 5% on various real-world datasets. Its two-stage architecture—comprising a lightweight CNN-based pose estimation model and a temporal smoothing mechanism—ensures stability across frames even in the presence of partial occlusion or erratic movements. This makes it particularly suitable for medical activity recognition where subtle pose changes are crucial (Laiba and Ahmad, 2024).

**FIGURE 4:**
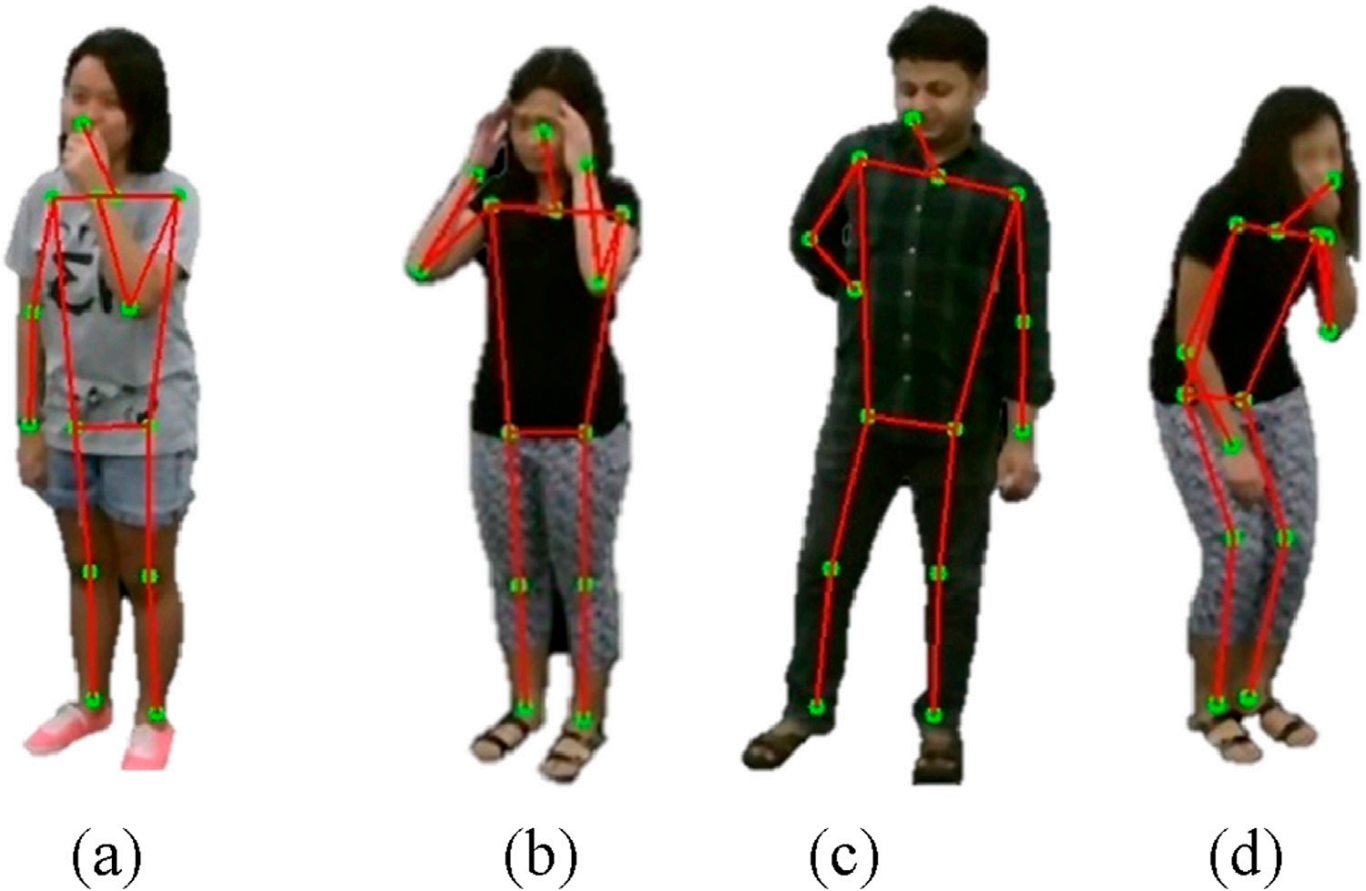
Illustration of Skeleton Generation on RGB frames **(a)** Cough **(b)** Headache **(c)** Backache **(d)** Vomit on NTU RGB + D 120 dataset.

The MediaPipe Pose model served as the framework of choice because it detects joints and generates precise skeletal models. Although the model initially identifies 33 landmarks across the human body, a subset of these points was selected to emphasize major joints, including the head, shoulders, elbows, wrists, hips, knees, and ankles. To enhance the skeletal model, a computed “neck” point was added as the midpoint between the left and right shoulder landmarks. This point was mathematically calculated using in Equation 13:

$$Neck\left(x,y\right)=\left(\frac{x_{11}+x_{12}}{2},\frac{y_{11}+y_{12}}{2}\right)$$ (13)

where $x_{11}$ and $x_{12}$ represent the x-coordinates of the left and right shoulders, respectively, and similarly for the y-coordinates (Muhammad et al., 2024). Following the identification of key landmarks, skeletal lines were drawn between specific pairs of points to create a structured representation of the human body. These connections, linking joints such as the neck to the shoulders, elbows to wrists, and hips to knees, were defined by the expression in Equation 14:

$$S\left(x_{1},y_{1},x_{2},y_{2}\right)=\left(\left(x_{1},y_{1}\right)\to \left(x_{2},y_{2}\right)\right)$$ (14)

where $S\left(x_{1},y_{1},x_{2},y_{2}\right)$ represents a line connecting two key points. The visualization method showed the human skeletal framework through a technique that overlaid the generated skeleton onto original silhouette images. The annotated silhouettes shown in Figure 4 represent human poses in a format that enables further analysis.

**FIGURE 5:**
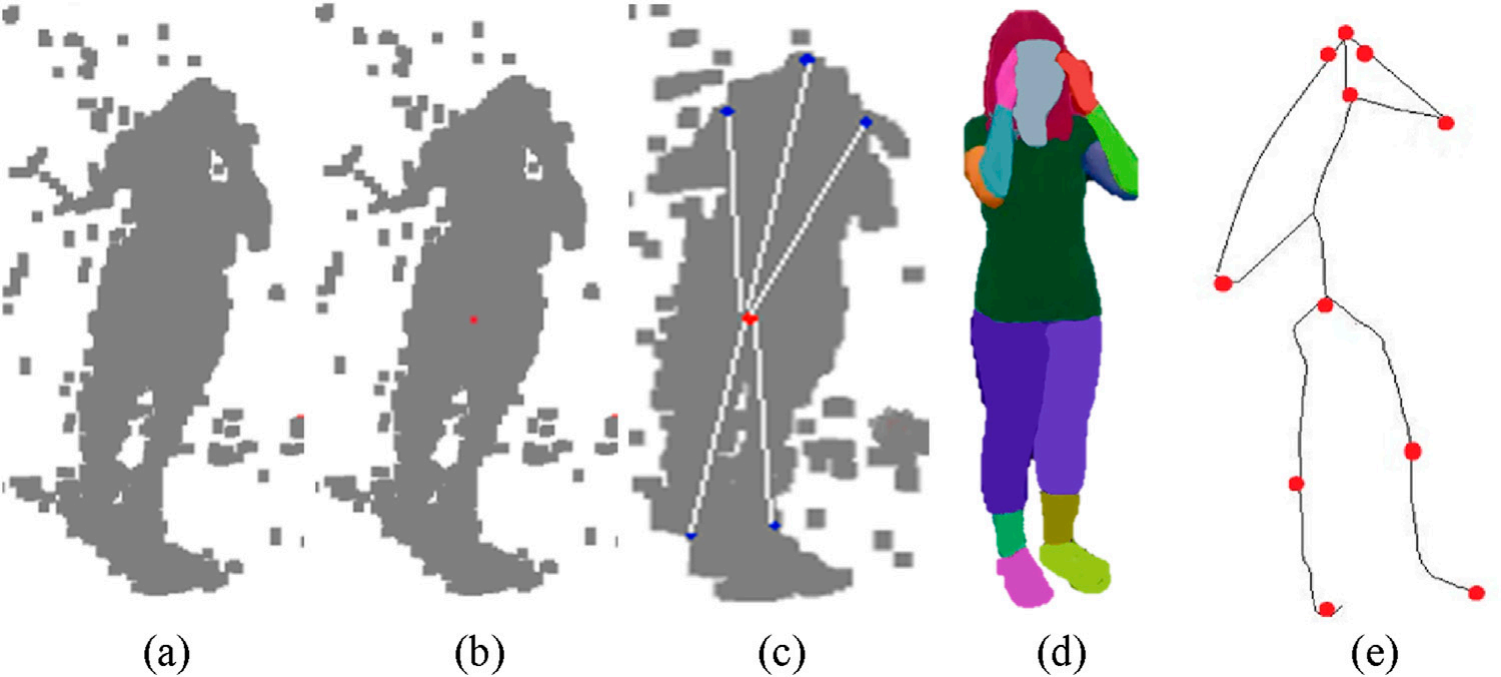
Illustration of Skeleton Generation on RGB frames **(a)** 3D Point Cloud **(b)** Torso Detection **(c)** Geodesic Distance **(d)** Body Part Segmentation **(e)** Skeleton Generation on NTU RGB + D 120 dataset.

The method developed by Handrich and Al-Hamadi (2015) provides an effective way to identify human body poses from depth images. The methodology builds a graph representation from 3D point clouds to compute distance measurements using geodesic paths while maintaining body pose independence. The system fits a rigid 3D torso model to the point cloud data to extract surface points before processing. Dijkstra’s algorithm computes geodesic paths to these points using edge weights defined by Euclidean distances between neighboring points (Saleha and Ahmad, 2024). Specifically, the shortest geodesic distance $g\left(a,b\right)$ ) between two graph nodes a and b is defined in Equation 15:

$$g\left(a,b\right)=\sum_{E\in P\left(a,b\right)}w\left(E\right)$$ (15)

where $w\left(E\right)$ is the edge weight, and $P\left(a,b\right)$ is the path connecting the nodes. The algorithm detects limb end points one by one according to their highest geodesic distance from the torso center. Unique detection is ensured by adding a zero-weight edge between the detected maximum and an intermediate point which requires distance recalculation. The body part segmentation is performed by labeling geodesic paths based on their relation to the torso center and the extremities of the body surface (Aljuaid et al., 2023). Finally, a kinematic skeleton model is adapted to the segmented body parts, fitting its joints to the 3D points by minimizing residual errors. Figure 5 represents the step-by-step graphical representation of the proposed technique.

### 3.5 Feature extraction

Feature extraction represents an essential building block for human interaction recognition (HMIR) systems that employ RGB and depth data. The system uses an integrated technique to acquire powerful spatial and temporal features. The analysis of motion dynamics and spatial relationships for RGB data relies on Kinetic Energy features, Histogram of Optical Flow (HOF), angular geometric features and eight round angles. Depth data processing uses Random Occupancy Patterns (ROP) in conjunction with 2.5D point clouds and Movement polygons to model global body structures and depth information (Aftab Ahmad et al., 2024). The system achieves enhanced activity recognition accuracy through its combined use of different methods which capture localized body movements while also detecting full-body spatial-temporal patterns.

#### 3.5.1 Kinetic energy features

Human Motion Intention Recognition (HMIR) detects detailed complicated movements through kinetic energy features to recognize medical conditions involving coughing sneezing and falling alongside headache and neck pain. Medical assessments of localized and global body movements need the tracking of both linear and rotational body segment motions. Medical condition detection reaches high accuracy by using kinetic energy features to identify physical motion strength levels and pattern distribution throughout various body motions (Mujtaba and Ahmad, 2024). The total kinetic energy $E_{total}$ for the human body is calculated by summing the contributions from both linear and rotational motions of all body segments and is given as Equation 16:

$$E_{total}=\sum_{i=1}^{n}\left(\frac{1}{2}m_{i}v_{i}{\left(t\right)}^{2}+\frac{1}{2}I_{i}\omega_{i}{\left(t\right)}^{2}\right)$$ (16)

where n represents the number of body segments, $m_{i}$ is the mass of the i−th segment, $v_{i}\left(t\right)$ is its linear velocity, $I_{i}$ is the moment of inertia, and $\omega_{i}\left(t\right)$ is its angular velocity at time t . The first term, linear kinetic energy $\frac{1}{2}m_{i}v_{i}{\left(t\right)}^{2}$ , captures the translational motion of body parts, which is particularly useful for identifying conditions such as falling or rapid upper body jerks during coughing and sneezing. The linear velocity $v_{i}\left(t\right)$ can be expressed as Equation 17:

$$v_{i}\left(t\right)=\sqrt{\dot{x}{\left(t\right)}^{2}+\dot{y}{\left(t\right)}^{2}+\dot{z}{\left(t\right)}^{2}}$$ (17)

where $\dot{x}{\left(t\right)}^{2}+\dot{y}{\left(t\right)}^{2}+\dot{z}{\left(t\right)}^{2}$ are the temporal derivatives of the spatial coordinates of the body segment. During falling motions the body segments experience high linear velocities, but coughing and sneezing activities involve strong linear displacements focused on the torso along with the neck and shoulder areas (Laiba and Ahmad, 2024). The second term in the expression describes rotational kinetic energy $\frac{1}{2}I_{i}\omega_{i}{\left(t\right)}^{2}$ which calculates angular motion energy around joints and becomes essential for detecting head tilts during headaches and minimal neck rotations related to neck pain. The angular velocity $\omega_{i}\left(t\right)$ is calculated as Equation 18:

$$\omega_{i}\left(t\right)=\sqrt{\dot{\theta }{\left(t\right)}^{2}+\dot{\phi }{\left(t\right)}^{2}+\dot{\psi }{\left(t\right)}^{2}}$$ (18)

where $\dot{\theta }{\left(t\right)}^{2}+\dot{\phi }{\left(t\right)}^{2}+\dot{\psi }{\left(t\right)}^{2}$ represent the derivatives of rotational angles about the three axes. The moment of inertia $I_{i}$ , which depends on the mass and geometry of the segment, is given by Equation 19:

$$I_{i}=k_{i}m_{i}r_{i}^{2}$$ (19)

where $k_{i}$ is a shape-dependent constant and $r_{i}$ is the distance between the axis of rotation and the center of mass of the segment. The rotational energy output from neck movements with pain or delicate head tilts concentrates primarily in upper body segments but the energy expenditure from falling distributes more heavily between multiple segments (Amir et al., 2020b). Kinetic energy features enable a comprehensive body dynamics analysis by measuring both linear and rotational energy patterns throughout all body segments to establish exact medical diagnosis distinctions. Stage seven shows how kinetic energy features are illustrated through visual diagrams to depict energy changes which occur across frames (Bonato et al., 2024).

**FIGURE 6:**
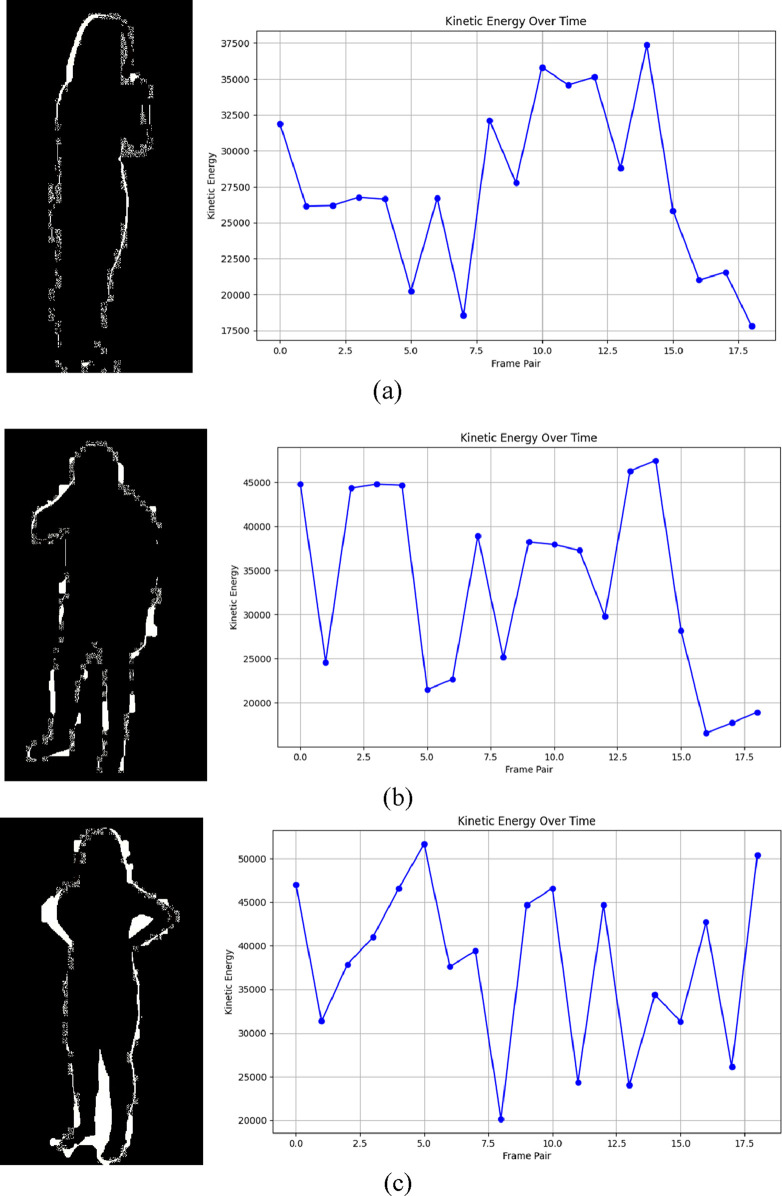
Illustration of Kinetic Energy over time on RGB frames **(a)** Cough **(b)** Neckache **(c)** Backache on NTU RGB + D 120 dataset.

Medical condition recognition benefits strongly from kinetic energy features because these features detect both forceful activities including falls and small bodily movements beyond basic position detection capabilities (Iqra et al., 2025). Changes in kinetic energy levels across multiple frames produce active motion profiles that help spot brief occurrences such as sneezes and coughs. Medical monitoring systems benefit from kinetic energy features because these metrics demonstrate both accuracy under uncertain sensor data and resistance to minor measurement errors. Figure 6 illustrates results of kinetic energy features (Liu et al., 2021).

#### 3.5.2 Histogram of optical flow (HOF)

The human interaction recognition system employs HOF features to detect fundamental movements related to medical conditions and minor actions including coughing and sneezing and falls as well as head movements and neck pain. Pixel-level movement tracking operated by HOF creates exact temporal representations of moving patterns preserving detailed motion data (Nazar and Jalal, 2025). The features show particular success in recognizing activities linked to medical conditions because they effectively handle abrupt together with gradual motion changes.

**FIGURE 7:**
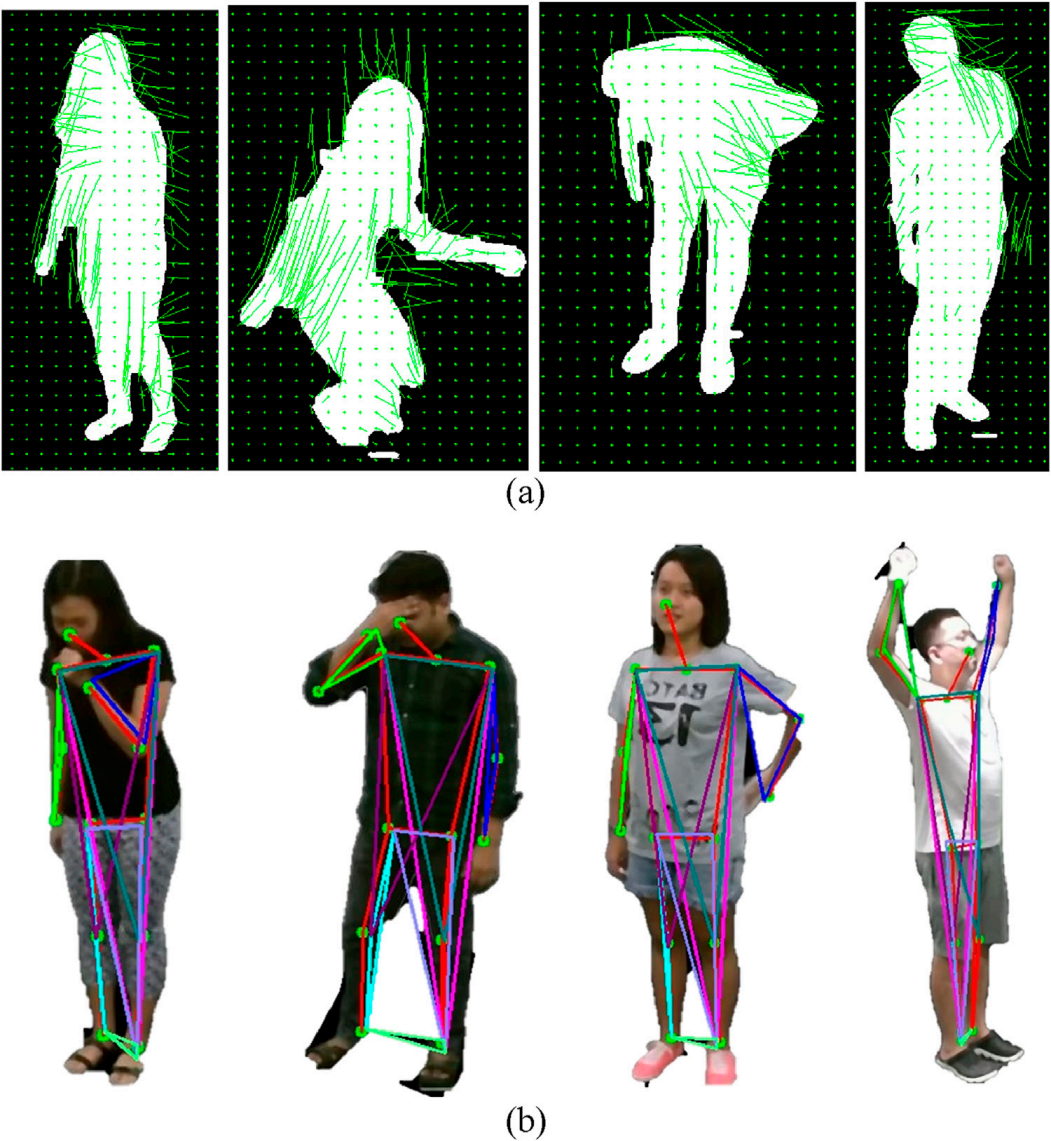
Illustration of HOF and Angular Geometric Features on RGB frames **(a)** Staggering Falling Down Backache Stretch Oneself on NTU RGB + D 120 dataset. Illustration of Histogram of Optical Flow (HOF) on RGB frames **(b)** Staggering Falling Down Vomit Backache on NTU RGB+D 120 dataset.

The optical flow at each pixel is represented as a vector $\left(u,v\right)$ , where u and v denote the horizontal and vertical components of motion between consecutive frames. To construct the Histogram of Optical Flow, the motion vectors are first divided into a fixed number of directional bins $\theta_{k}$ , where $\theta_{k}=\arctan\left(\frac{v}{u}\right)$ represents the angle of motion for each pixel. The magnitude of motion, $=\sqrt{u^{2}+v^{2}}$ , is used to weight the contribution of each motion vector to the corresponding bin (Mujtaba et al., 2025). The HOF descriptor for a given region is computed as Equation 20:

$$H_{k}=\sum_{p\in R}M_{p}\cdot \delta \left(\theta_{p}\in bin\;k\right)$$ (20)

where $H_{k}$ represents the cumulative weighted contribution to the k−th bin, p denotes the pixels in the region R , $M_{p}$ is the magnitude of motion at pixel p , and $\delta \left(\theta_{p}\in bin\;k\right)$ is an indicator function that assigns the motion vector to its respective bin. A histogram develops during this procedure to represent dominant motion directions as well as their strength within the analyzed region. The extracted HOF features appear in Figure 7.

#### 3.5.3 Angular geometric features

The quantification of skeletal point angular relationships serves as an essential factor for HMIR through angular geometric features. The system derives these features from essential body landmarks which enable the capture of human body structure and geometric alignment throughout activities. The analysis of angular relationships between predefined body segment sets through angular geometric features generates a robust motion dynamic and postural representation that helps identify medical conditions through specific physical activities (Rafiq and Jalal, 2025).

Extraction of skeletal points during earlier processing enables researchers to create meaningful triangular geometric structures linking joint relationships. Multiple triangles in this model emerge when researchers choose three relevant body landmarks starting from shoulder-elbow-wrist to hip-knee-ankle. For instance, triangles such as (11,13,15) and (23,25,27) represent upper limb and lower limb configurations, respectively, while (11,29,12) and (23,29,24) capture torso and hip alignments. The arranged triangles measure the entire human body position space to reveal essential details about regional and complete body movements (Laiba and Jalal, 2025).

The calculation of angles at triangle vertices allows researchers to measure body segment connections. Given three points $P_{1}$ , $P_{2}$ and $P_{3}$ , where $P_{2}$ is the vertex of interest, the angle is computed using the vectors $\vec{v_{1}}=P_{1}-P_{2}$ and $\vec{v_{2}}=P_{3}-P_{2}$ . The angle θ is determined as Equation 21:

$$\theta =\arccos\left(\frac{\vec{v_{1}}\cdot \vec{v_{2}}}{\left\|\vec{v_{1}}\right\|\left\|\vec{v_{2}}\right\|}\right)$$ (21)

where $\vec{v_{1}}\cdot \vec{v_{2}}$ represents the dot product of the vectors, and $\left\|\vec{v_{1}}\right\|\left\|\vec{v_{2}}\right\|$ are their magnitudes. This algorithm determines the exact angular position between linked body segments to identify critical movement characteristics including flexion and extension and rotational alignment. The extracted angular geometric features appear in Figure 7.

#### 3.5.4 Eight round angles

The eight round angles use a powerful spatial encoding technique which documents directional adjustments according to the 8 Freeman Chain Code principles. The quantification of position and direction relationships through spatial features remains essential for HMIR because these features show how body positions modify human silhouette structure and orientation. This method tracks skeletal posture modifications by analyzing both curvatures and directional shifts across skeletal features which creates a universal spatial motion description (Ashraf et al., 2025).

The system starts by utilizing skeletal points from previous steps that align with important joints together with body landmarks. The points extracted from the human body silhouette function as references for silhouette outline creations which are used as inputs in the 8 Freeman Chain Code algorithm. The algorithm represents the curve by dividing the space around each skeletal point into eight equally spaced angular sectors, the eight principal compass directions: 0°, 45°, 90°, 135°, 180°, 225°, 270°, and 315°. Each curve point receives directional assignments through its relative neighbor position which generates a sequence of directional codes (Mohammed et al., 2025).

**FIGURE 8:**
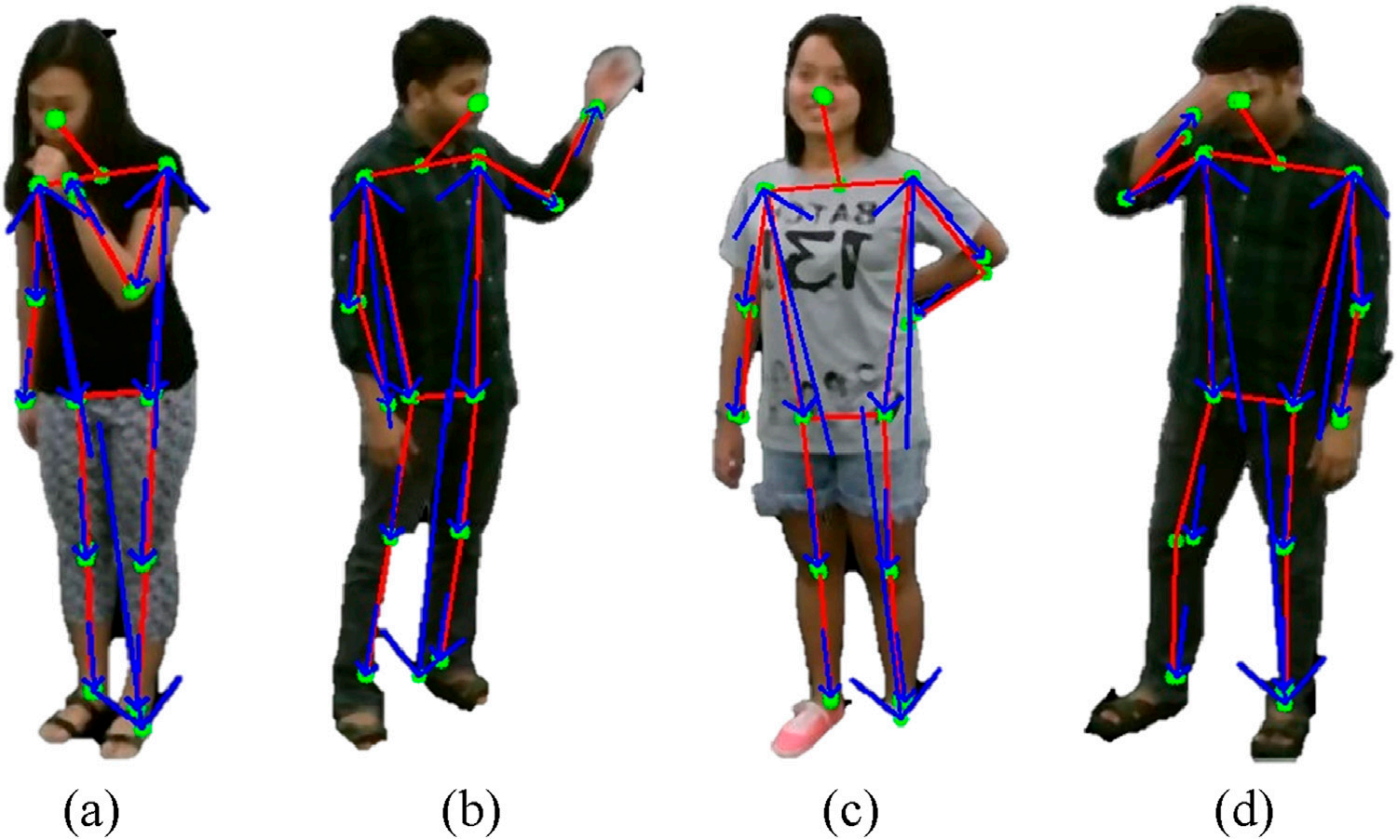
Illustration of Eight Round Angles on RGB frames **(a)** Cough **(b)** Fanself **(c)** Backache **(d)** Headache on NTU RGB + D 120 dataset.

For a given boundary point *b* with *n* contour points, the curve $C_{b}$ is defined as a series of connected points from the starting point $C_{0}$ to the endpoint $C_{n-1}$ . The algorithm moves clockwise along the curve, measuring the directional changes at each step. For example, if the direction changes from $C_{0}$ to $C_{1}$ , the next step is to measure the transition from $C_{1}$ to $C_{2}$ , and so on, until all points in the contour have been processed. The directional changes are expressed in Equation 22:

$$∆\theta =\theta_{i+1}-\theta_{i}$$ (22)

where $\theta_{i}$ and $\theta_{i+1}$ represent the angular direction of successive points along the contour. The encoding system produces angular characteristics which summarize skeletal outline modifications (Naif et al., 2025). These eight round angles obtained from the process deliver essential data about spatial movement and posture relationships. The directional code measurements indicate sudden jerky movements through large code changes while smoother code transitions detect controlled and refined actions that include head movements and postures. The computed features demonstrate high discrimination power and their translation and scaling invariance and tolerance of minor noise make them ideal for HMIR applications in medical environments. Figure 8 illustrates these features (Goecks et al., 2022).

#### 3.5.5 2.5-D point clouds

2.The 5D point cloud system provides an effective approach to extract depth features which support HMIR applications in medical settings for coughing, sneezing, falling and back and neck pain assessment (Sumbul et al., 2025). The single-perspective view of 2.5D point clouds captures body surface depth while maintaining efficient spatial representation when compared to the complete coverage needed in 3D point clouds. The representation works well with medical HMIR applications because it maintains critical motion details with minimal processing complexity and data redundancy.

**FIGURE 9:**
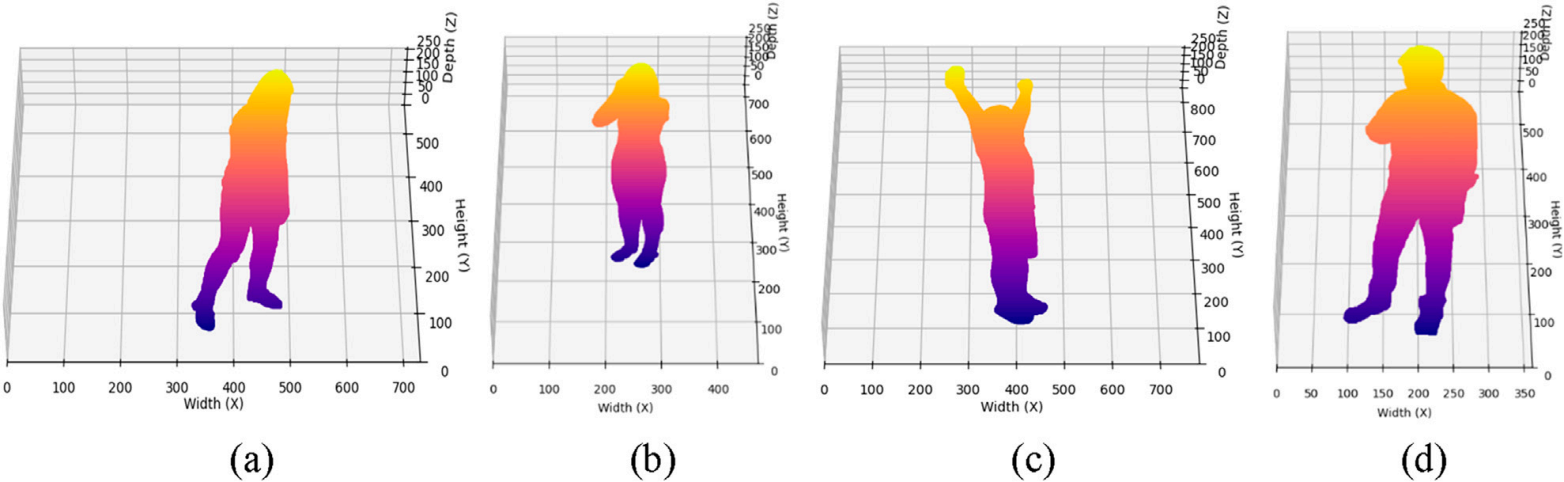
Illustration of 2.5D Point Cloud on Depth frames **(a)** Staggering **(b)** Cough **(c)** Stretch Oneself **(d)** Chest Pain on NTU RGB + D 120 dataset.

The depth map $D\left(x,y\right)$ , representing the distance of each point $\left(x,y\right)$ from the sensor, is transformed into a 2.5D point cloud $P={\left\{\left(x_{i},y_{i},z_{i}\right)\right\}}_{i=1}^{N}$ , where $z_{i}=D\left(x_{i},y_{i}\right)$ is the depth value. To capture the dynamic behavior of body movements, features such as velocity, acceleration, and curvature of the point trajectories are computed over successive frames (Awan et al., 2024). For example, the velocity $v\left(t\right)$ of a point is given in Equation 23:

$$v\left(t\right)=\frac{P\left(t\right)-P\left(t-∆t\right)}{∆t}$$ (23)

where ∆t is the time interval between frames. Additionally, the curvature $\kappa \left(t\right)$ , which provides insights into local surface deformation due to movement, is calculated using Equation 24:

$$\kappa \left(t\right)=\frac{\left\|v\left(t\right)\times a\left(t\right)\right\|}{{\left\|v\left(t\right)\right\|}^{3}}$$ (24)

where $a\left(t\right)$ is the acceleration. The system differentiates between activities that show different spatial and temporal motion patterns through these features (Zahra et al., 2025). The human body’s respiratory motions which include coughing and sneezing activate head and torso region high-frequency local deformations but extensive body movements occur during a fall. The analysis of these characteristics throughout the 2.5D point cloud space enables successful detection and classification of subtle medical condition-related movements. The compact nature of 2.5D point clouds makes them efficient for real-time HMIR systems in medical monitoring systems because they reduce computational complexity (Seerat et al., 2025). Figure 9 demonstrates 2.5 D point cloud results.

#### 3.5.6 Random occupancy pattern (ROP)

Random Occupancy Pattern (ROP) features effectively describe the spatiotemporal dynamics of HMIR while demonstrating particular performance in medical condition detection including coughing sneezing and falls and posture-induced pain (Wang et al., 2012; Li et al., 2017). The tracking of voxel occupancy patterns across time in a 3D voxel grid delivers robust motion representation and enables accurate activity recognition because ROP features demonstrate resistance to sensor variability and noise.

**FIGURE 10:**
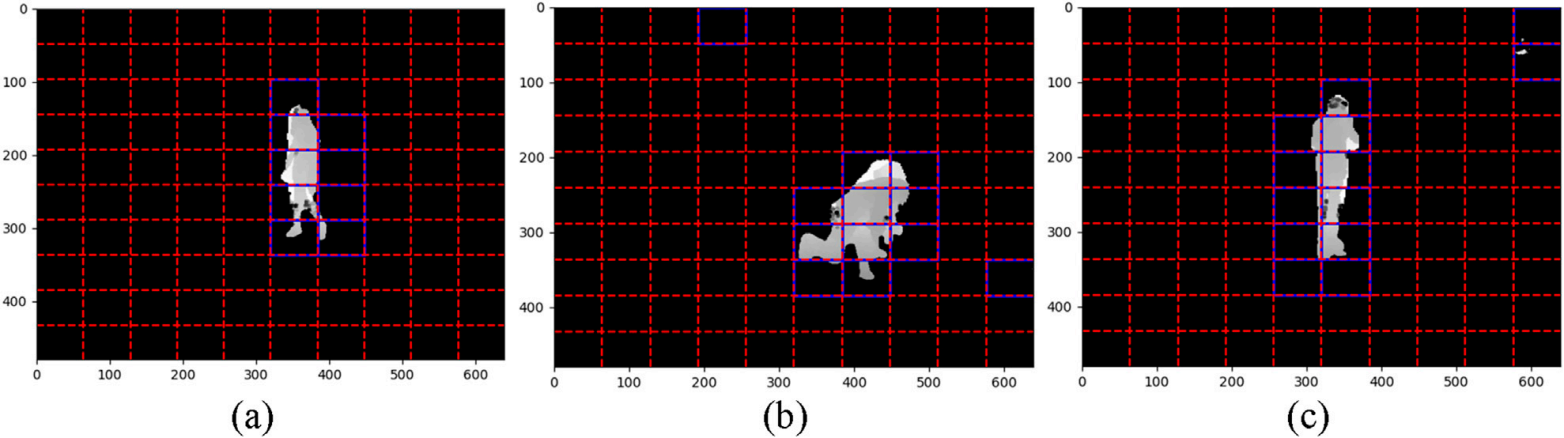
Illustration of ROP Features on Depth frames **(a)** Staggering **(b)** falling down **(c)** Chest Pain on NTU RGB + D 120 dataset.

The voxel grid is defined by partitioning the spatial domain into uniform cells, and the occupancy of each voxel at time t is denoted as $o_{i}\left(t\right)$ , where $o_{i}\left(t\right)=1$ if the voxel i is occupied and $o_{i}\left(t\right)=0$ otherwise. A random sampling approach is applied to select a subset of voxels, reducing computational cost while preserving discriminatory information (Muhammad et al., 2025). The ROP feature vector $f_{ROP}$ is then constructed by concatenating the occupancy patterns across the sampled voxels over T frames using Equation 25:

$$f_{ROP}=\left[o_{i}\left(t_{1}\right),o_{i}\left(t_{2}\right),\ldots o_{i}\left(t_{T}\right)\right],i\in S$$ (25)

where S is the set of randomly selected voxels. The effective activity differentiation under ROP occurs through analysis of spatial occupancy patterns. The speed of body motion in falling creates extensive grid occupancy changes but coughing or sneezing leads to localized changes in the torso and head region. The temporal variations of ROP features reveal movement velocity and repetition patterns to support medical diagnosis (Ahmed et al., 2023). The sparse sampling structure of ROP features optimizes computational resources and memory which makes them ideal for real-time medical surveillance. The resistance of ROP features to body size and orientation changes and environmental conditions enables reliable performance in multiple medical situations (Iqra and Jalal, 2025). The figure depicting ROP feature results appears in Figure 10.

#### 3.5.7 Movement polygons

Movement polygons serve as an effective skeleton-based feature extraction methodology that extracts information for HMIR analyses from depth sensor data (Tayyab and Jalal, 2025). Movement polygons display skeletal spatial motion dynamics through three-dimensional joint position projection to two-dimensional movement polygons. A set of three-dimensional skeletal joint positions $J={\left\{\left(x_{i},y,z_{i}\right)\right\}}_{i=1}^{N}$ forms the joint positions into a two-dimensional polygon. The boundary of this polygon is determined, and its centroid, $G=\left(G_{x},G_{y}\right)$ , is calculated as Equations 26, 27:

$$G_{x}=\frac{1}{N}\sum_{i=1}^{N}x_{i}$$ (26)

$$G_{y}=\frac{1}{N}\sum_{i=1}^{N}y_{i}$$ (27)

**FIGURE 11:**
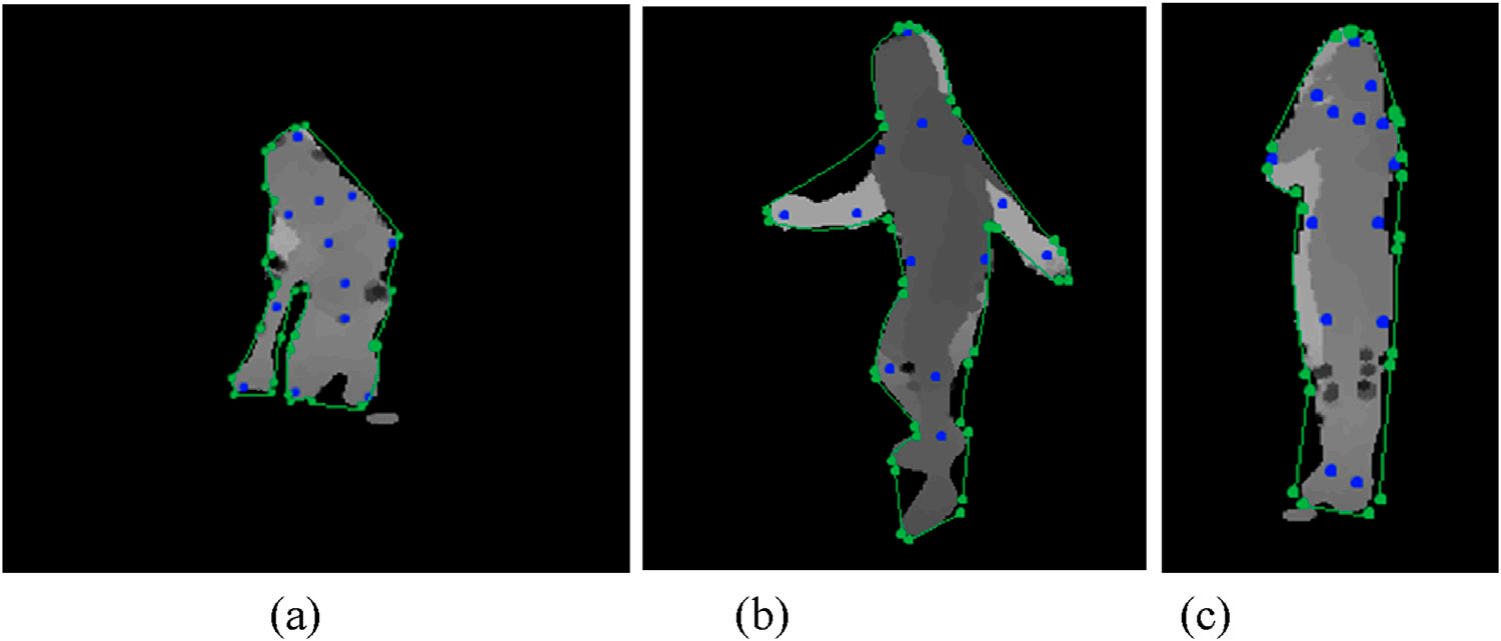
Illustration of 2.5D Point Cloud on Depth frames **(a)** Falling Down **(b)** Staggering **(c)** Headache on NTU RGB + D 120 dataset.

To reduce the dimensionality of the data, the distance of each polygon boundary point from its centroid is computed as Equation 28:

$$d_{i}=\sqrt{{\left(x_{i}-G_{x}\right)}^{2}+{\left(y_{i}-G_{y}\right)}^{2}}$$ (28)

where $d_{i}$ represents the distance of the i−th boundary point. These distances are then sampled at uniformly spaced angles, resulting in a one-dimensional feature vector $={\left\{d\left(\theta \right)\right\}}_{\theta =1}^{360}$ . This 1D feature vector captures the geometric characteristics of skeletal movement, enabling efficient representation and discrimination of human actions (Tayyab et al., 2025). In addition to the polygon boundary features, the trajectory of the most-moving joint over time is considered to model temporal dynamics. The most-moving joint is discovered by calculating the covariance matrix of its coordinates across frames. Its angular displacement, $\tan^{-1}\left(\frac{y\left(t\right)-y\left(0\right)}{x\left(t\right)-x\left(0\right)}\right)$ , is tracked across successive frames to produce a 20-dimensional trajectory vector. The final feature vector combines the polygonal boundary features and the most-moving joint trajectory, yielding a compact representation suitable for classification (Saleha et al., 2025). These features are highly effective for HMIR, as they capture both spatial and temporal aspects of motion while maintaining computational efficiency. Figure 11 illustrates movement Polygons.

Below is the proposed summary Table 1 that outlines the effects of each feature described in Section 3 of your manuscript. This table provides a concise yet informative overview of the modalities, motion characteristics, and contributions of each feature type to the HMIR task:

**TABLE 1:** Summary of feature effects on human motion intention recognition.

Feature type	Modality	Description & captured characteristics	Contribution to HMIR performance
Kinetic Energy Features	RGB	Quantifies linear and rotational body motion via velocity and angular velocity	Enhances detection of forceful and high-energy movements (e.g., falling, coughing, sneezing)
Histogram of Optical Flow (HOF)	RGB	Measures pixel-wise motion direction and magnitude over time	Captures temporal movement flows, beneficial for classifying staggered or jerky activities
Angular Geometric Features	RGB	Computes joint angles between skeletal landmarks	Differentiates postural changes (e.g., neck pain vs. back pain) based on geometric articulation
Eight Round Angles	RGB	Encodes silhouette contour direction using Freeman Chain Code	Offers shape-based cues for pose transitions, robust to scale/rotation
2.5D Point Clouds	Depth	Projects depth maps into spatially aligned surface representations	Provides detailed spatial structure for complex actions, resistant to occlusion
Random Occupancy Pattern (ROP)	Depth	Tracks voxel-wise occupancy across space and time	Distinguishes global vs. local activity spread and intensity
Movement Polygons	Depth	Constructs 2D polygonal projections of joint trajectories with centroid analysis	Captures motion symmetry, body coordination, and repetitive patterns (e.g., stretching, waving)

### 3.6 Feature optimization via stochastic gradient descent

After extracting robust full-body and point-based features, these features are concatenated to form a comprehensive feature vector. However, the resulting feature vector resides in a high-dimensional space, which introduces challenges related to computational efficiency and system performance. To address this, dimensionality reduction through feature optimization techniques is applied, ensuring improved computational efficiency and enhanced system performance (J. Ahmad et al., 2025).

In the proposed architecture, Stochastic Gradient Descent (SGD) is employed as the optimization algorithm. SGD is a highly efficient technique for training machine learning models, particularly suitable for large-scale datasets and complex parameter spaces. Unlike traditional gradient descent methods, which compute parameter updates based on the gradient of the loss function over the entire dataset, SGD updates the model parameters iteratively using individual training examples or small mini batches of data (Laiba and Jalal, 2025; Laiba et al., 2025). This iterative approach is computationally efficient and accelerates convergence, particularly for high-dimensional optimization problems. The parameter update rule for SGD is formalized as Equation 29:

$$\theta_{t+1}≔\theta_{t}-\eta \;\nabla_{\theta }L\left(\theta_{t};x_{i},y_{i}\right)$$ (29)

where $\theta_{t}$ represents the parameter vector at iteration *t*, η>0 denotes the learning rate, and $\nabla_{\theta }L\left(\theta_{t};x_{i},y_{i}\right)$ is the gradient of the loss function *L* with respect to the parameters θ , computed for the training example $\left(x_{i},y_{i}\right)$ . To generalize this formulation for a mini-batch of size *m*, the gradient is computed as the mean of the gradients over the batch, expressed as Equation 30:

$$\theta_{t+1}≔\theta_{t}-\eta \frac{1}{m}\sum_{i=1}^{m}\nabla_{\theta }L\left(\theta_{t};x_{i},y_{i}\right)$$ (30)

**FIGURE 12:**
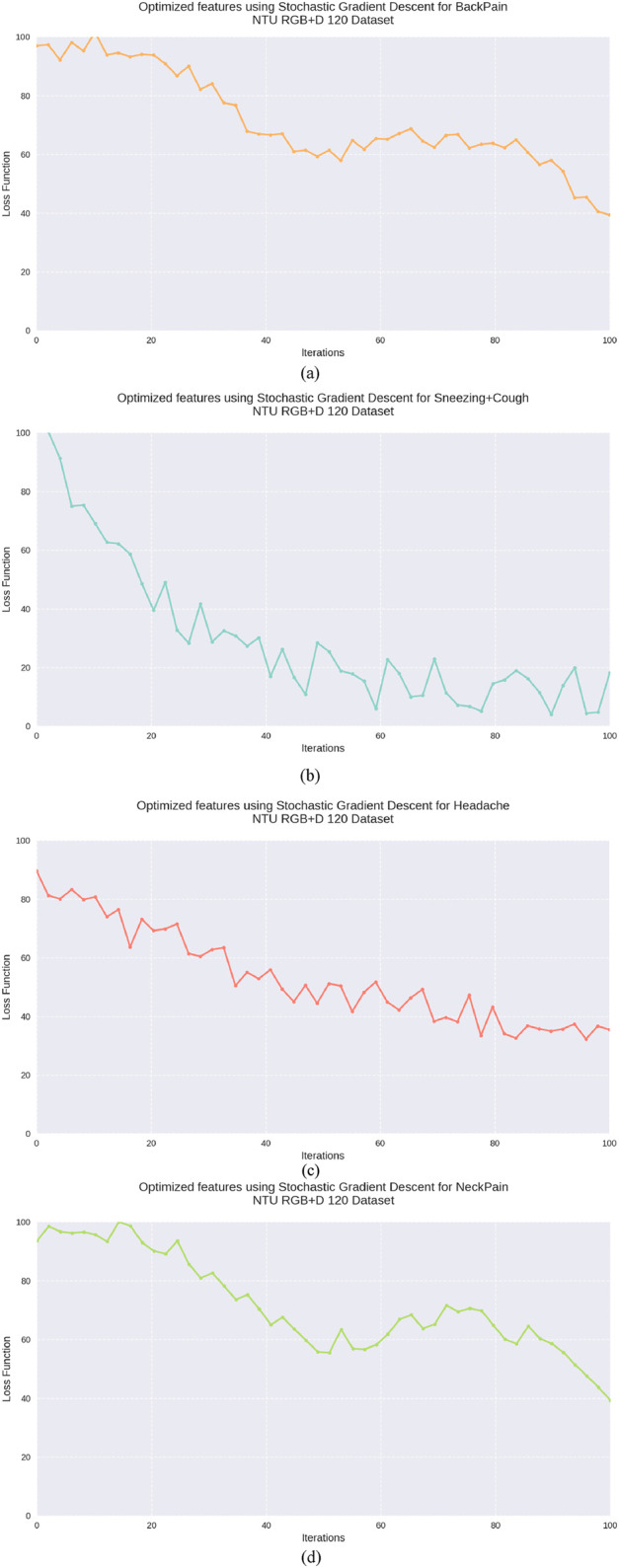
Graphical Representation of Stochastic Gradient Descent **(a)** Back Pain **(b)** Sneezing + Cough **(c)** Headache **(d)** Neck Pain on NTU RGB + D 120 dataset.

The utilization of SGD for feature optimization not only reduces the dimensionality of the feature vector but also facilitates faster convergence to optimal parameter values, even in the presence of large-scale and high-dimensional data (M. Javeed et al., 2024). Furthermore, the stochastic nature of the updates introduces noise into the optimization process, which can help the model escape local minima and converge towards better global minima in non-convex loss landscapes. Figure 12 illustrates SDG results.

### 3.7 Feature classification via deep neuro fuzzy classifier

The classification task was performed using the Deep Neuro-Fuzzy Classifier, a hybrid model that integrates the learning capabilities of neural networks with the interpretability of fuzzy logic systems. This approach leverages the strengths of both paradigms to achieve robust performance on complex datasets (Israr and Ahmad, 2024; Wang et al., 2019).

**FIGURE 13:**
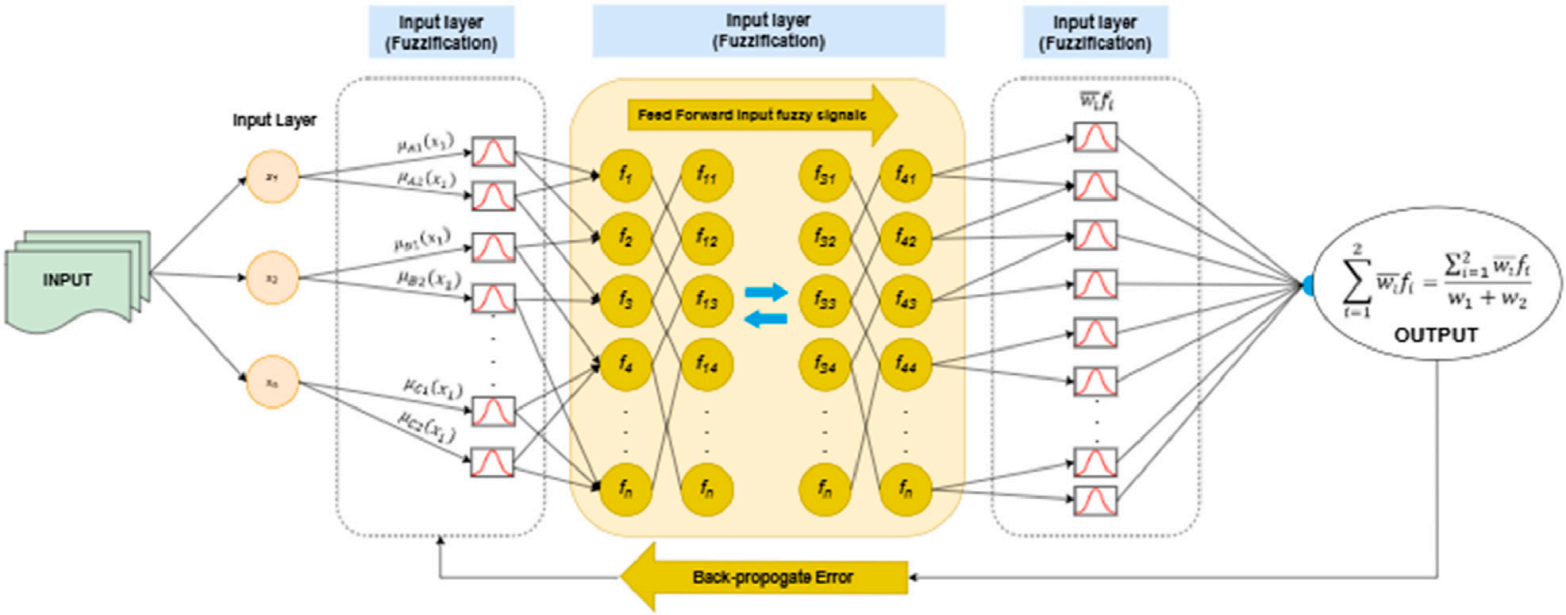
Architecture of Deep neuro Fuzzy Classifier.

The classifier operates by mapping input features into fuzzy membership values, which are then processed through neural layers for adaptive learning. Let the input feature vector be represented as $x={\left[x_{1},x_{2},\ldots ,x_{n}\right]}^{T}\in R^{n}$ .The system utilizes fuzzy membership functions to compute the degree of membership for each feature using Equation 31:

$$\mu A_{i}\left(x_{i}\right)=\frac{1}{1+{\left|\frac{x_{i}-c_{i}}{\sigma_{i}}\right|}^{2m}}$$ (31)

where $\mu A_{i}\left(x_{i}\right)$ is the membership degree of $x_{i}$ to fuzzy set $A_{i}$ , $c_{i}$ is the center of the fuzzy set, $\sigma_{i}$ is the spread parameter, and m>1 is the fuzzification factor. These membership values are then passed through a set of fuzzy rules of the form:

$$R_{k}∶IF\;x_{1}\;is\;A_{1}^{k}\;AND\;x_{2}\;is\;A_{2}^{k}\ldots THEN\;y_{k}=w_{k}$$ (32)

where in Equation 32 $k=1,2,...,K,A_{j}^{k}$ represents the fuzzy set associated with the *j*th input for the *k*th rule, and $w_{k}$ is the output weight corresponding to $R_{k}$ . The aggregated output of the fuzzy inference system is computed using the weighted average in Equation 33:

$$y=\frac{\sum_{k=1}^{K}\mu_{k}\cdot w_{k}}{\sum_{k=1}^{K}\mu_{k}}$$ (33)

where $\mu_{k}=\prod_{i=1}^{n}\mu_{A_{i}^{k}}\left(x_{i}\right)$ is the rule activation strength. To optimize the system, the neural network component adjusts the parameters, $c_{i}$ , $\sigma_{i}$ and $w_{k}$ using backpropagation (Zhang et al., 2021). The loss function employed for training is typically the mean squared error (MSE) in Equation 34:

$$L=\frac{1}{N}\sum_{i=1}^{N}\left(y_{i}-{\hat{y}}_{i}\right)$$ (34)

where N is the total number of samples, $y_{i}$ is the actual output, and ${\hat{y}}_{i}$ is the predicted output. The deep architecture further enhances the model’s performance by incorporating multiple layers of rule-based transformations, allowing for hierarchical feature extraction (Tayyab et al., 2024; Li et al., 2021). The method’s multi-layered structure enables advanced pattern learning within an interpretable framework that works well for medical condition assessment applications. Figure 13 shows the architecture of deep neuro fuzzy Classifier.

#### 3.7.1 Hyperparameter tuning

To optimize system performance, we performed empirical tuning of key hyperparameters in both the feature optimization and classification stages using grid search on the NTU RGB + D 120 validation set. For the Stochastic Gradient Descent (SGD) algorithm, we evaluated learning rates in the range (0.001, 0.01, 0.05, 0.1) and selected 0.01 as optimal for convergence speed and stability. Mini-batch sizes were tested in (16, 32, 64), with 32 providing the best trade-off between gradient stability and training time. The maximum number of epochs was set to 100 with early stopping criteria based on validation loss stagnation for 10 consecutive epochs.

For the Deep Neuro-Fuzzy Classifier, we fine-tuned the number of fuzzy rules in the range (5, 10, 20), fuzzification factor in (1.5, 2.0, 2.5), and Gaussian membership function spread parameter σ ∈ [0.2, 1.0]. Optimal performance was achieved with 10 rules, a fuzzification factor of 2.0, and σ = 0.5. Regularization weight was set to 0.01 to prevent overfitting, and the classifier was trained using the Adam optimizer with an initial learning rate of 0.001. All hyperparameters were selected based on average performance across 5-fold cross-validation to ensure robustness and generalizability.

## 4 Performance evaluation

The proposed system was rigorously assessed using three benchmark datasets: NTU RGB + D, PKU-MMD, and UWA3DII. The system underwent complete performance evaluation using confusion matrices and precision, recall metrics and F1 scores. Multiple evaluation metrics showed that the system performed exceptionally well to reach its defined goals.

The evaluation took place on a Windows 11 64-bit platform using an Intel Xeon processor and 32 GB memory with an Intel i7 11th Gen CPU that featured 8 cores. The implemented system optimized its computation pipelines for efficient dataset handling and real-time processing needs.

### 4.1 Dataset description

#### 4.1.1 NTU RGB + D 120 dataset

**FIGURE 14:**
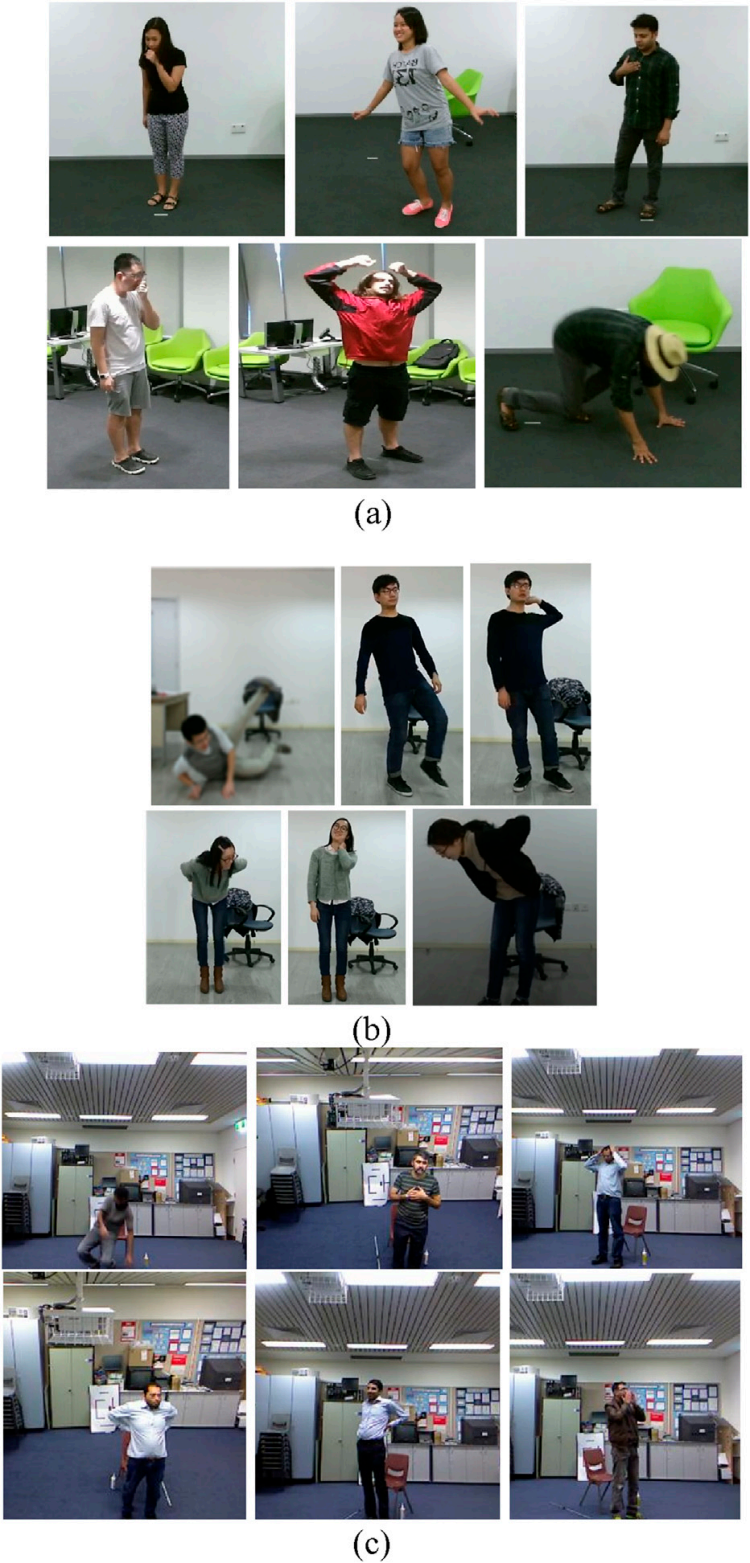
Some examples frames of **(a)** NTU RGB + D 120 **(b)** PKUMMD **(c)** UWA3DII dataset.

The NTU RGB + D 120 dataset operates as the leading action recognition benchmark that supports research into both fundamental body actions and health-related body movements. The dataset contains 114,480 video samples showing 120 different action categories performed by 40 individuals. The dataset classifies actions into three main groups: The NTU RGB + D dataset comprises 82 daily activities such as face wiping and coin tossing alongside 12 health-specific actions including sneezing and neck pain incidents and 26 interactive actions that include punching and hugging and kicking. A total of three camera positions were used to record actions at −45°, 0° and +45° horizontal angles to expand viewpoint diversity. Notable for its multi-modal nature, the dataset includes depth information, 3D skeletal joint data, RGB video frames, and infrared sequences, making it especially valuable for medical condition monitoring and patient care research. Illustration of some interactions in Figure 14a.

#### 4.1.2 PKU-MMD dataset

The PKU-MMD dataset is a large-scale human action analysis dataset with a focus on health-related behavior recognition and multi-modality. Captured by the Kinect v2 sensor, it has two phases of data collection. Phase 1 has 1,076 video sequences from 51 action classes, with 66 subjects captured from three camera views. The dataset contains close to 20,000 action instances, consisting of over 5.4 million frames, with a mean video duration of 3–4 min at 30 FPS. Actions are categorized into 41 daily activities (waving, drinking, etc.) and 10 interactive behaviors (hugging, handshakes, etc.). Of specific interest to rehabilitation and health, the dataset captures both daily activity and important interactions crucial to the study of patient-to-patient and patient-to-caregiver communication. Its structured design enables precise evaluation of motion patterns vital for healthcare applications. Illustration of some interactions in Figure 14b.

#### 4.1.3 UWA3DII dataset

The UWA3DII dataset was developed to enhance HMIR by incorporating diverse motion patterns and multi-view. Thirty movements including walking while holding the chest and sneezing and falling comprise the dataset which includes ten participants. Each action was recorded four times using four different views: The dataset presents views from both the front and left, right and top surfaces. The continuous Kinect sensor data collection has produced a dataset with natural motion variation which serves medical purposes for fall detection and movement disorder assessment. Clinical medical research benefits from this dataset because self-occlusions combined with action similarities create processing complexity. Illustration of some interactions in Figure 14c.

## 5 Results and analysis

The experimental section is organized into five comprehensive analyses to evaluate the effectiveness and robustness of the proposed multi-modal HMIR framework. Initially, confusion matrix analysis is conducted across the NTU RGB + D 120, PKU-MMD, and UWA3DII datasets to provide a detailed breakdown of classification performance across multiple medical activity classes. This is followed by the computation of key evaluation metrics including precision, recall, and F1-score, offering insights into the model’s balance between sensitivity and specificity. To further assess the training dynamics and generalization capability, a learning curve analysis is presented, illustrating the relationship between training and validation loss over multiple epochs. An ablation study is then performed to quantify the contribution of each core module and feature types such as preprocessing, segmentation, skeletal modeling, and both RGB and depth-based features—by systematically removing each component and evaluating its impact on overall classification accuracy. Finally, the proposed model is benchmarked against several state-of-the-art approaches using standardized datasets, demonstrating its superior performance in terms of classification accuracy, robustness to occlusion, and adaptability to complex human motion patterns. This experimental organization ensures a comprehensive and objective evaluation of the proposed framework’s capabilities in real-world rehabilitation monitoring applications.

### 5.1 Experiment 1: confusion matrix

The first experiment shows confusion matrix results for both datasets. The confusion matrix shows a simple graphical view of the classifier’s performance as it shows both successful and unsuccessful classification instances per class. Tables 2–4 present the confusion matrix for NTU RGB + D 120, PKU-MMD and UWA3DII Dataset.

**TABLE 2:** Confusion matrix calculated over the NTU RGB + D 120 Dataset.

Classes	SC	STG	FD	HA	CP	BP	NP	NUS	FS	YWN	SO	BN
SC	**94**	1	1	1	1	1	0	0	0	1	0	0
STG	1	**93**	1	1	1	1	0	0	0	2	0	0
FD	1	1	**94**	2	1	0	0	0	0	1	0	0
HA	0	0	1	**96**	1	0	0	0	0	2	0	0
CP	1	0	1	0	**90**	3	1	1	1	2	0	0
BP	0	0	1	0	1	**91**	3	2	1	1	0	0
NP	0	0	0	0	0	2	**94**	2	1	1	0	0
NUS	0	0	0	0	0	0	2	**95**	1	2	0	0
FS	0	0	0	0	0	0	0	1	**95**	2	1	1
YWN	0	0	0	0	1	1	0	0	0	**95**	1	1
SO	0	0	0	0	0	0	0	0	0	1	**96**	2
BN	0	0	0	0	0	0	0	0	0	0	2	**98**
Mean Accuracy = 94.50%												

SC = Sneezing + Cough, STG = staggering; FD = falling down; HA = headache; CP = chest pain; BP = BackPain, NP = NeckPain, NUS = nausea; FS = fanself; YWN = yawn; SO = stretch oneself; BN = blow nose. Bold values in the confusion matrix displays recognition accuracy for individual class.

**TABLE 3:** Confusion matrix calculated over PKU MMD Dataset.

Classes	FL	RTHT	TB	TC	TH	TN	UF	WF
FL	**92**	1	2	0	1	2	1	1
RTHT	1	**87**	1	2	1	0	0	8
TB	3	2	**85**	1	3	2	3	1
TC	0	0	0	**97**	0	0	0	3
TH	3	2	1	3	**85**	2	1	2
TN	0	0	1	0	1	**97**	1	0
UF	0	1	1	0	1	0	**96**	1
WF	0	4	2	2	3	0	0	**89**
Mean Accuracy = 91.23%								

FL, falling; RTHT rub two hands together; TB, touch back; TC, touch chest; TH, touch head; TN, touch neck; UF, use fan; WF, wipe face. Bold values in the confusion matrix displays recognition accuracy for individual class.

**TABLE 4:** Confusion matrix calculated over UWA3DII - Dataset.

Classes	FD	CH	HD	BA	IW	LD	SN	CG
FD	**90**	2	2	2	1	1	1	1
CH	2	**85**	2	2	1	2	2	5
HD	0	4	**82**	4	1	1	4	4
BA	0	2	1	**95**	0	0	1	1
IW	2	1	1	0	**85**	3	2	6
LD	3	0	1	0	0	**95**	1	0
SN	0	2	2	1	0	1	**92**	2
CG	1	3	2	4	1	1	2	**86**
Mean Accuracy = 88.60%								

FD, falling down; CH, chest pain; HD, headache; BA, backache; IW, irregular walking; LD, laying down; SN, sneezing; CG, coughing. Bold values in the confusion matrix displays recognition accuracy for individual class.

### 5.2 Experiment 2: Precision, recall and F1 score

**FIGURE 15:**
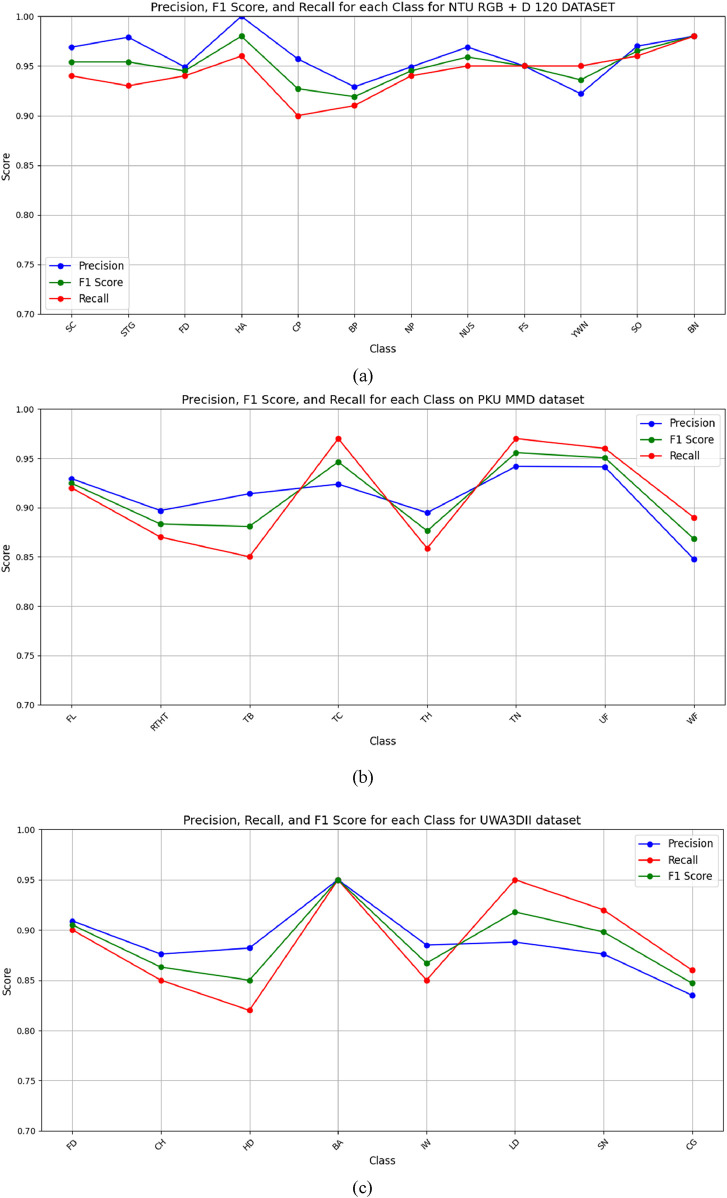
Line graph for Precision, Recall, and F1 score for **(a)** NTURGB+D120, **(b)** PKUMMD and **(c)** UWA3DII Datasets.

The F1 score, recall and precision metrics were represented through line graphs in Figure 15 across the NTU RGB + D 120, PKU MMD and UWA3DII datasets. These visualizations provide a performance comparison of the proposed model which demonstrates its effectiveness across multiple datasets alongside various performance benchmarks.

#### 5.2.1 Discussion and analysis

Experimental findings demonstrate that the proposed Multi-Modal Vision Sensor Framework successfully identifies human interactions. The framework combines RGB and depth data through state-of-the-art feature extraction methods while using stochastic gradient descent optimization and a deep neuro-fuzzy classifier. The proposed model delivers landmark accuracy results of 94.50% on NTU RGB + D 120% and 91.23% on PKUMMD and 88.60% on UWA3DII.

Upon examining the confusion matrices, we can observe that actions such as falling down, coughing, and sneezing achieve high classification accuracy since they have very unique motion patterns. Nevertheless, there do exist some activities, particularly ones with fine-grained or overlapping movements, such as neck pain and back pain, with mild misclassifications. This occurs due to the similarity in skeletal movement and the challenge of discriminating against fine-grained actions in complex situations.

The precision, recall, and F1 scores further validate the system’s reliability, with consistently high performance across all datasets. The feature extraction techniques, particularly kinetic energy and angular geometric features, significantly contribute to differentiating motion dynamics in human activities. Additionally, the use of depth-based features like 2.5D point clouds and random occupancy patterns enhances robustness against environmental variations such as occlusions and lighting changes.

Despite the promising results, some limitations persist. Occlusion handling and real-time adaptability remain areas for improvement, especially in highly dynamic environments. Future work can explore the integration of self-attention mechanisms, adaptive feature selection, and transformer-based architectures to further refine the classification process.

### 5.3 Experiment 3: learning curve analysis

**FIGURE 16:**
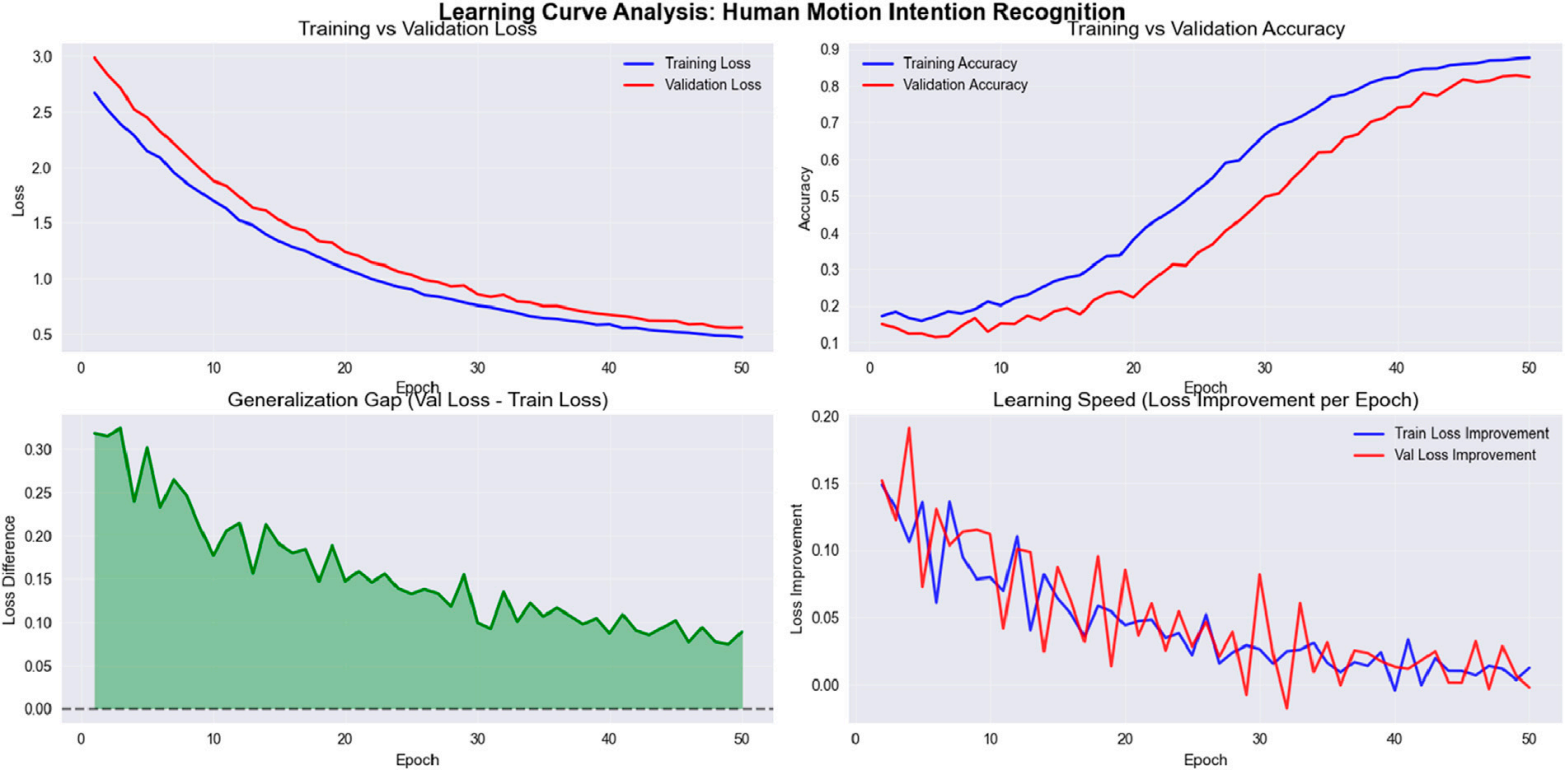
Line graph for Precision, Recall, and F1 score for NTU RGB + D 120, PKU MMD and UWA3DII Datasets.

To assess the convergence behavior and generalization capacity of the proposed model, we present the learning curve in Figure 16. The graph plots training and validation loss over successive epochs during training on the NTU RGB + D 120 dataset. As observed, the training loss steadily decreases while the validation loss follows a similar trend with minimal divergence. This indicates that the model avoids overfitting and maintains generalization across unseen samples.

The learning curve exhibits several noteworthy characteristics. First, both training and validation losses decrease rapidly during the initial epochs (1–15), demonstrating efficient parameter optimization. This is followed by a more gradual reduction phase (epochs 15–35), where the model fine-tunes its parameters. Finally, the curves stabilize in the later epochs (35–50), suggesting convergence to an optimal solution.

The minimal gap between training and validation losses, particularly in the later stages of training, confirms the model’s ability to generalize effectively. This robust generalization is especially significant given the multimodal nature of the NTU RGB + D 120 dataset, which contains complex action patterns across 120 different classes performed by multiple subjects.

The smooth convergence curve also confirms the effectiveness of the feature optimization via stochastic gradient descent (SGD) with momentum. The consistent descent in both curves without significant oscillations demonstrates that our selected learning rate and batch size provide stable optimization dynamics. Furthermore, the fuzzy membership functions effectively capture the inherent variations in skeletal motion patterns, allowing the model to adapt to the multimodal distribution of action classes.

These results support the model’s reliability for large-scale, multimodal data and validate our architectural design choices. The convergence behavior suggests that the model has successfully learned discriminative features from skeletal data while avoiding both underfitting and overfitting issues that commonly plague deep learning approaches to action recognition.

### 5.4 Experiment 4: ablation study

To evaluate the relative contribution of each module and feature type used in the proposed Human Motion Intention Recognition (HIR) framework, we conducted an extensive ablation study across three benchmark datasets: NTU RGB + D 120, PKUMMD, and UWA3DII. This study involved systematically removing (one at a time) each major component in the feature extraction and processing pipeline and measuring the resulting classification accuracy.

Table 5 summarizes the results. The baseline (“Full Model”) includes all components: Preprocessing (PR), Skeleton and Key Point Generation (SG), Silhouette Segmentation (SS), Kinetic Energy (KE), Histogram of Optical Flow (HOF), Angular Geometric Features (AGF), Eight Round Angles (ERA), 2.5D Point Clouds (PC), Random Occupancy Patterns (ROP), and Movement Polygons (MP). Each subsequent experiment removes one component while keeping the others intact (denoted as “WT”—Without).

**TABLE 5:** Ablation study results (accuracy %).

Experiment	PR	SS	SG	KE	HOF	AGF	ERA	PC	ROP	MP	NTU RGB D 120	PKUMMD	UWA3DII
Full Model	✔	✔	✔	✔	✔	✔	✔	✔	✔	✔	**94.50**	**91.23**	**88.60**
WT PR	**✖**	✔	✔	✔	✔	✔	✔	✔	✔	✔	92	90	86
WT SS	✔	**✖**	✔	✔	✔	✔	✔	✔	✔	✔	90	88	84
WT SG	✔	✔	✖	✔	✔	✔	✔	✔	✔	✔	88	86	83
WT KE	✔	✔	✔	✖	✔	✔	✔	✔	✔	✔	92	89	86
WT HOF	✔	✔	✔	✔	✖	✔	✔	✔	✔	✔	91	86	85
WT AGF	✔	✔	✔	✔	✔	✖	✔	✔	✔	✔	90	88	86
WT ERA	✔	✔	✔	✔	✔	✔	✖	✔	✔	✔	92	89	86
WT PC	✔	✔	✔	✔	✔	✔	✔	✖	✔	✔	92	87	84
WT ROP	✔	✔	✔	✔	✔	✔	✔	✔	✖	✔	91	88	85
WT MP	✔	✔	✔	✔	✔	✔	✔	✔	✔	✖	91	88	85

WT, without; PR, preprocessing; SS, silhouette segmentation; SG, skeleton generation; KE, kinetic energy; HOF, histogram of optical flow; AGF, angular geometric features; ERA, eight round angels; PC, 2.5 D Point Cloud, ROP, random occupancy pattern; MP, movement polygons. Bold values in the Ablation study displays mean recognition accuracy against each dataset.

### 5.5 Experiment 5: comparisons with state of the art (SOTA)

To validate the superiority of our proposed system, we compare its performance against state-of-the-art (SOTA) models in human interaction recognition. Table 6 presents a comparative analysis of classification accuracy across NTU RGB + D 120, PKUMMD, and UWA3DII datasets.

**TABLE 6:** Comparisons with state of the art using deep learning models.

Method	Accuracy %		
	NTU RGB + D 120	PKU MMD	UWA3DII
Song et al. (2016)	81.2	—	—
Lee et al. (2017)	74.60	—	—
Luvizon et al. (2018)	85.5	—	—
Song et al. (2018)	—	44.4	—
Li et al. (2017)	—	53.3	—
Li et al. (2017)	—	54.8	—
Vemulapalli and Chellappa (2016)	—	—	43.4
H. Rahmani et al. (2014)	—	—	52.2
Rahmani and Mian (2015)	—	—	76.9
Liu et al. (2017)	—	—	73.8
Zhang et al. (2019)	—	—	81.4
Proposed	**94.50%**	**91.23%**	**88.60%**

The results indicate that our method outperforms existing SOTA models across all datasets, achieving a significant accuracy improvement of 8%–15% compared to leading approaches. The fusion of multi-modal data, advanced feature engineering, and deep neuro-fuzzy classification contributes to this enhanced performance. Unlike recurrent neural networks (RNNs) or purely CNN-based approaches, our system integrates spatio-temporal features with interpretable fuzzy logic, making it well-suited for real-world applications in healthcare and assistive technologies (Song et al., 2018).

Future enhancements may involve fine-tuning fuzzy rule sets, incorporating transformer-based spatio-temporal processing, and optimizing model inference for edge-based applications to further improve performance and scalability.

## 6 Implication of proposed system

The proposed Multi-Modal Vision Sensor Framework demonstrates widespread utility across healthcare as well as surveillance applications while also serving rehabilitation purposes and assistive technology needs. The system achieves robust and accurate HMIR of complex actions alongside medical activities such as sneezing, coughing, back pain, and fainting through depth data integration with RGB information. Precise detection of minimal movements enables useful applications during patient monitoring and elderly care and rehabilitation treatments that rely on accurate medical activity recognition.

The system advances medical patient monitoring through real-time tracking because it identifies activities which indicate patient distress or discomfort. Its ability to distinguish postural discomfort and abnormal movements and falls makes this system appropriate for smart hospitals and home rehabilitation systems and wearable health monitoring systems. The system achieves better classification accuracy by employing advanced feature extraction methods together with the deep neuro-fuzzy classifier in difficult conditions involving occluded views or reduced visibility. The system provides dependable detection of medical priority activities which proves essential for both intervention effectiveness and prompt medical response.

The system extends its effects throughout security and surveillance operations. The system provides excellent capabilities for automated security solutions and workplace safety applications because it effectively detects emergency actions from standard activities. The integration of spatio-temporal features alongside multi-modal sensor data enhances real-world anomaly detection capabilities which creates improved public safety and incident response systems.

The framework demonstrates strong deployment potential because its high accuracy results emerged from multiple benchmark testing scenarios. The integration of aggressive feature extraction with optimized classification and multi-modal data fusion establishes solid groundwork for building future intelligent monitoring systems. Future work should enhance real-time adaptability and increase dataset diversity and implement transformer-based frameworks which improve recognition in dynamic complex environments.

## 7 Limitations

While the proposed multi-modal rehabilitation monitoring system demonstrates high accuracy and robustness across multiple datasets, it also presents certain limitations. Firstly, the system’s performance may degrade under severe occlusion or poor-quality depth sensing, which affects silhouette and skeleton extraction accuracy. Secondly, although we evaluated the framework across three diverse datasets, cross-dataset generalization may require further domain adaptation or fine-tuning, especially for unseen medical gestures or patient-specific behaviors. Additionally, the integration of multiple feature streams and the deep neuro-fuzzy classifier introduces computational overhead, which may limit real-time applicability in low-resource or embedded edge devices. Lastly, the system has not yet been tested in live clinical or at-home rehabilitation scenarios, which will be an essential next step for validating practical deployment and usability.

## 8 Conclusion

A novel and extensive approach to HMIR for medical applications was developed through joint RGB and depth analysis of the NTU RGB + D 120, PKUMMD, and UWA3DII datasets. The system includes advanced feature engineering methodologies which integrate kinetic energy alongside histogram of optical flow (HOF) features and angular geometric features along with eight round angles for RGB data and 2.5D cloud ROP features together with Movement polygons of depth data. A complete analysis of activity recognition emerges through the combination of SGD optimization with deep neuro-fuzzy classifier and the introduced features which enables high accuracy medical activity recognition. Experimental results demonstrate that the proposed framework proves both durable and versatile by producing excellent results across numerous datasets. This framework demonstrates both operational precision and computational effectiveness which makes it an attractive solution for practical medical assistance technologies.

## Data Availability

Publicly available datasets were analyzed in this study. This data can be found here: https://github.com/shahroudy/NTURGB-D; https://www.research.lancs.ac.uk/portal/en/datasets/uwa3dii-dataset-skeleton(33507bb7-e3e4-41c2-8175-4bf58f801c4f).html.
